# Temporal trends and health inequalities in global, regional, and national years lived with disability of severe periodontitis from 1990 to 2021

**DOI:** 10.1371/journal.pone.0337994

**Published:** 2026-02-02

**Authors:** Shuang Zhang, Si-Yu Liu, Qiong Wang, Ya-Xi Suo, Yue-Qin Zhang, Chuan-Yu Hu, Long Xie

**Affiliations:** 1 State Key Laboratory of Oral & Maxillofacial Reconstruction and Regeneration, Key Laboratory of Oral Biomedicine Ministry of Education, Hubei Key Laboratory of Stomatology, School & Hospital of Stomatology, Wuhan University, Wuhan, Hubei, China; 2 Department of Preventive Dentistry, School of Stomatology, Wuhan University, Wuhan, Hubei, China; 3 Department of Fifth Outpatient, The Affiliated Stomatological Hospital of Nanjing Medical University, Nanjing, Jiangsu, China; 4 Department of Stomatology, Huaihua hospital of traditional Chinese medicine, Huaihua, Hunan, China; 5 Department of Stomatology, Tongji Hospital, Tongji Medical College, Huazhong University of Science and Technology, Wuhan, Hubei, China; 6 School of Stomatology, Tongji Medical College, Huazhong University of Science and Technology, Wuhan, Hubei, China; 7 Hubei Province Key Laboratory of Oral and Maxillofacial Development and Regeneration, Wuhan, Hubei, China; Shahid Beheshti University of Medical Sciences School of Dentistry, IRAN, ISLAMIC REPUBLIC OF

## Abstract

This study evaluates changes in cross-national disparities in the burden of severe periodontitis between 1990 and 2021. All data on severe periodontitis used in this study were derived from the 2021 Global Burden of Disease (GBD) study. Annual years lived with disability (YLDs) and their estimated annual percentage changes (EAPCs) were calculated and stratified by year, age, geographical region, and socio-demographic index (SDI) at global, regional, and national levels. Decomposition analysis assessed the contributions of demographic and epidemiological factors to the evolving burden of severe periodontitis. A frontier analysis identified areas for improvement and disparities among countries based on development levels. Distributional inequalities were measured using the slope index of inequality (SII) and concentration index. The autoregressive integrated moving average model (ARIMA) was used to project the disease burden up to 2036. In 2021, there were 6,903.28 thousand YLDs (95% uncertainty interval [UI]: 2,772.28−14,106.18 thousand) attributed to severe periodontitis globally. Between 1990 and 2021, the global age-standardized rate (ASR) of YLDs showed a stable trend, increasing slightly from 79.62 (95% UI: 31.46–169.62) to 80.89 (95% UI: 32.47–165.37) with an EAPC of 0.08% (95% confidence interval [CI]: −0.03 to 0.18). Population growth accounted for 66.73% of the global increase in YLDs. SII values rose from 12.72 (95% CI: 1.92–23.52) in 1990 to 44.99 (95% CI: 31.14–58.85) in 2021, while the concentration index decreased from 0.05 (95% CI: −0.04 to 0.13) in 1990 to 0.035 (95% CI: −0.06 to 0.13). According to the forecasts, the global ASR of YLDs for severe periodontitis is projected to show a slight decline over the next 15 years. Significant potential exists for reducing the burden of severe periodontitis across countries, irrespective of their development levels. Severe periodontitis remains a significant global health challenge, with substantial cross-country disparities that persist despite overall stable trends in global YLDs. Targeted interventions and policies are urgently needed to address these disparities, focusing on improving oral health outcomes across all countries, regardless of their socio-demographic development levels.

## Introduction

Periodontitis is a multifactorial chronic inflammatory condition driven by microbial dysbiosis within dental plaque biofilms. It triggers a dysregulated host immune-inflammatory response, leading to the progressive destruction of periodontal support structures, including the ligament and alveolar bone. Several factors influence its pathogenesis, including genetic predisposition, environmental factors such as smoking and inadequate oral hygiene, and systemic conditions like diabetes mellitus and obesity [1,2]. If untreated, periodontitis can progress to severe forms, resulting in significant oral and systemic complications such as tooth loss, impaired mastication, and diabetes-related complications [3,4].

Beyond the oral cavity, periodontitis has systemic implications. Its chronic inflammatory nature and bacteremia contribute to the development of cardiovascular diseases, diabetes, adverse pregnancy outcomes, and other systemic conditions [5,6]. For instance, poorly controlled diabetes increases susceptibility to severe periodontitis, whereas effective periodontal treatment can improve glycemic control [6]. Moderate to severe periodontitis has been linked to an elevated risk of all-cause mortality and cardiovascular disease-related mortality among adults with diabetes [7]. Other associations include respiratory diseases, rheumatoid arthritis, and an increased risk of certain cancers, underlining the widespread health impacts of periodontitis [8].

A high disease burden is typically associated with sociodemographic underdevelopment, limited access to healthcare, and the poor performance of healthcare systems [9]. In contrast, recent studies suggest that regions or countries with higher socio-economic status may also face an unusually high burden of these diseases [10]. The economic implications are also substantial, with annual direct treatment costs exceeding $3.49 billion in the United States and €156.12 billion in Europe as of 2018 [11]. This difference highlights the intricate interaction of factors like lifestyle modifications and genetic predisposition, which together influence the development and advancement of these conditions. Therefore, monitoring the trends of severe periodontitis in different countries is essential for improving resource distribution, and reducing the disease burden and its associated complications.

The GBD database provides a robust framework for assessing the burden of diseases, including severe periodontitis, using standardized metrics such as YLDs. These metrics quantify the non-fatal burden of diseases by integrating prevalence and severity data, offering insights into chronic conditions like periodontitis that impair quality of life without directly causing mortality [12]. Typically, high-income countries report lower burdens due to better access to oral healthcare, while low-income countries face higher untreated rates due to resource limitations [13]. Highlighting disparities in severe periodontitis across regions and countries is critical for optimizing resource allocation and addressing global health inequalities. These studies play a crucial role in enhancing the judicious distribution of health resources [14].

This study advances beyond previous GBD-based analyses of severe periodontitis in several important ways. First, it integrates multiple analytic frameworks-decomposition, frontier, and WHO-recommended health inequality metrics-into a single comprehensive assessment, enabling us to disentangle demographic and epidemiological drivers, identify efficiency gaps relative to socio-demographic development, and quantify both absolute and relative disparities over time. Second, we explore the non-linear relationship between SDI and disease burden, identifying threshold effects that have not been documented in the periodontitis literature. Third, we provide forward projections to 2036 using ARIMA modelling, offering policy-relevant foresight for resource allocation and preventive strategies. These methodological innovations allow our work to go beyond descriptive trend updates and generate actionable insights for oral health policy-making at global, regional, and national levels.

## Methods

### Data source

The data of severe periodontitis for this study, including years lived with disability (YLDs) and their respective 95% uncertainty intervals (UI), were extracted from the Global Burden of Disease (GBD) 2021 database (accessible at [http://ghdx.healthdata.org/gbd-result-tool]). In the GBD 2021, the case definition of periodontitis is “Community Index of Periodontal Treatment Needs (CPITN) Class IV, attachment loss (AL) >6 mm, or gingival pocket depth (PD) > 5mm” [15]. These definitions correspond to International Classification of Diseases, Ninth Revision (ICD-9) codes 523.0–523.9 and ICD-10codes K05.0-K05.6. To enlarge the dataset, severe periodontitis cases identified using alternative criteria-such as CPITN Class III, AL > 5 mm, or AL > 4 mm-were harmonized with the reference definitions via the MR-BRT meta-regression approach, which accounts for and corrects systematic biases arising from varying case definitions [16].

The dataset spans the period from 1990 to 2021 and encompasses 21 GBD regions and 204 countries and territories, each assigned a unique SDI [17]. This extensive dataset provides a detailed perspective on the burden of severe periodontitis across diverse geographic and socioeconomic landscapes [18]. Global trends in periodontitis were analyzed across the following age groups: 15–19, 20–24, 25–29, 30–34, 35–39, 40–44, 45–49, 50–54, 55–59, 60–64, 65–69, 70–74, 75–79, 80–84, 85–89, 90–94, and 95 + years.

### Statistical analysis

#### Burden description.

The burden of severe periodontitis across 21 GBD regions and 204 countries and territories was quantified using the total number of YLDs and the age-standardized rate (ASR) of YLDs. Together, these metrics provide a comprehensive overview of the global impact of the disease. To highlight regional disparities in the burden of severe periodontitis, global maps were created to visualize the geographic distribution of ASR of YLDs for the year 2021. The SDI, a composite measure of socio-economic development, was employed to explore its correlation with health outcomes. The SDI integrates fertility rates, education levels, and income indicators [19]. The Spearman rank correlation coefficient was used to quantify the relationship between the ASR of YLDs and the SDI. Ethics approval and participant consent were not applicable for this study as the data used were obtained from a publicly available database.

### Trend analysis

Global, regional, and national trends in the ASR of YLDs attributable to severe periodontitis were assessed using the estimated annual percentage change (EAPC). The EAPC was calculated based on a regression model fitted to the natural logarithm of the ASR over time: ln(ASR)=α+β·year+e. Here, β represents the slope of the regression line. The EAPC was subsequently derived using the formula: EAPC=(exp(β)−1)×100 [20]. A negative EAPC value indicates a decreasing trend, whereas a positive value signifies an increasing trend. This approach provides a robust framework for assessing temporal changes in the global burden of severe periodontitis.

### Decomposition analysis

To further investigate the drivers of changes in severe periodontitis cases over time, decomposition analysis was performed based on the method proposed by Cheng *et al*. [21]. This technique dissects changes in the number of cases into three contributing factors: population growth, shifts in age structure, and changes in age-specific rates. The methodology involved evaluating the contribution of each factor individually, while keeping the other two factors constant.

### Frontier analysis

Frontier analysis was utilized to assess the relative efficiency of countries and regions in managing the burden of severe periodontitis in relation to their socio-economic development status (SDI). This analysis employed data envelopment analysis (DEA) to evaluate the efficiency of health systems in reducing the ASR of YLDs across different SDI levels. By identifying best-performing regions and highlighting inefficiencies, frontier analysis provides actionable insights into potential areas for improvement in public health strategies and resource allocation to mitigate the burden of severe periodontitis [22].

### Cross-country health inequality analysis

To examine cross-country inequalities in the burden of severe periodontitis, total YLDs and crude YLD rates were analyzed using two inequality metrics recommended by the World Health Organization: the Slope Index of Inequality (SII) and the Concentration Index [23]. Slope index of inequality was calculated by regression of the country-level YLDs due to severe periodontitis in all age populations on the sociodemographic development-related relative position scale, defined by the midpoint of the cumulative class range of the population ranked by SDI. SII provided an absolute measure of inequality, where positive values indicate a greater burden in higher-SDI countries, while negative values suggest a higher burden in lower-SDI countries. The Concentration Index assessed relative inequality by fitting a Lorenz concentration curve to the cumulative distribution of YLDs and population, ranked by SDI, with values closer to −1 indicating concentration in lower-SDI countries and values closer to 1 indicating concentration in higher-SDI countries.

### Predictive analysis

The autoregressive integrated moving average (ARIMA) model is a commonly applied statistical approach for predicting future trends in time series data [24]. To forecast the future burden of severe periodontitis over the next 15 years, we used the autoregressive integrated moving average model (ARIMA) in R.

All statistical analyses and graphics were performed using R version (version 4.3.3). Statistical significance was defined as a p-value less than 0.05.

## Results

### Global trends

Globally, the number of YLDs attributable to severe periodontitis increased from 3,620.52 thousand (95% uncertainty interval [UI]: 1,421.41−7,719.69 thousand) in 1990–6,903.28 thousand (95% UI: 2,772.28−14,106.18 thousand) in 2021, representing a 90.67% rise over the 32-year period (Table 1). The ASR of YLDs showed a slight increase from 79.62 per 100,000 population (95% UI: 31.46–169.62) in 1990 to 80.89 per 100,000 population (95% UI: 32.47–165.37) in 2021, demonstrating a stable trend with an estimated annual percentage change (EAPC) of 0.08% (95% confidence interval [CI]: −0.03 to 0.18) (Table 1). This near-zero EAPC indicates that the age-standardized burden of severe periodontitis has remained essentially unchanged over the past three decades.

**Table 1:** YLDs of severe periodontitis in 1990 and 2021 and its estimated annual percentage change from 1990 to 2021.

Location	1990		2021		EAPC % (95% CI) 1990–2021
	Number (×103, 95% UI)	Age-standardized YLDs per 100 000 population (95% UI)	Number (×103, 95% UI)	Age-standardized YLDs per 100 000 population (95% UI)	
Global	3,620.52(1,421.41−7,719.69)	79.62(31.46-169.62)	6,903.28(2,772.28−14,106.18)	80.89(32.47-165.37)	0.08(−0.03 to 0.18)
Male	1,864.55(730.73−3,981.69)	82.86(32.83-176.70)	3,489.13(1,398.74−7,146.19)	83.02(33.28-169.77)	0.04(−0.06 to 0.14)
Female	1,755.97(692.15−3,738.00)	76.51(30.29-162.82)	3,414.15(1,373.55−6,962.50)	78.88(31.70-161.22)	0.11(0.01 to 0.22)
**SDI**					
High SDI	675.04(267.68−1,436.74)	66.41(26.42-141.98)	1,021.2(406.50−2,047.77)	65.47(26.30-132.99)	−0.18(−0.38 to 0.02)
High-Middle SDI	730.38(288.35−1,576.85)	69.35(27.67-149.66)	1,231.03(492.70−2,492.76)	68.73(27.55-139.24)	0.20(0 to 0.41)
Middle SDI	1,047.10(414.76−2,236.76)	78.52(31.20-167.75)	2,249.56(909.68−4,517.59)	79.88(32.32-160.93)	0.23(0.10 to 0.35)
Low-Middle SDI	802.43(324.10−1,660.45)	96.49(38.29-198.97)	1,735.97(705.99−3,546.78)	98.50(39.88-199.41)	−0.01(−0.06 to 0.05)
Low SDI	362.25(143.52-746.67)	111.43(44.04-227.49)	660.69(268.31−1,356.34)	86.99(35.40-176.97)	−1.12(−1.28 to −0.95)
**Region**					
Andean Latin America	20.64(8.53-43.15)	75.24(30.60-156.62)	49.78(19.36-104.95)	75.28(29.58-158.55)	0.18(0.11 to 0.26)
Australasia	11.78(4.54-26.13)	53.03(20.48-117.45)	25.61(10.18-54.01)	64.45(25.05-136.61)	0.93(0.75 to 1.12)
Caribbean	29.33(11.79-60.67)	98.79(39.96-204.03)	45.96(18.35-95.06)	88.59(35.16-183.65)	−0.29(−0.41 to −0.18)
Central Asia	34.57(13.70-74.91)	65.68(26.10-142.10)	58.38(23.00-122.81)	60.29(23.69-125.58)	−0.30(−0.51 to −0.09)
Central Europe	84.53(33.15-183.76)	58.83(23.07-128.25)	107.88(42.68-219.15)	64.88(25.80-134.29)	0.42(0.35 to 0.49)
Central Latin America	107.63(43.07-223.98)	93.72(38-194.92)	246.59(99.48-506.79)	92.56(37.33-190.51)	0.06(0.03 to 0.10)
Central Sub-Saharan Africa	38.06(15.19-77.26)	111.25(44.62-228.84)	51.55(19.95-112.51)	61.21(23.79-132.14)	−2.62(−3.06 to −2.18)
East Asia	744.69(295.68−1,609.37)	71.47(28.52-154.70)	1,466.66(578.10−2,997.15)	69.55(27.77-143.33)	0.42(0.06 to 0.79)
Eastern Europe	201.22(81.00-424.93)	75.66(30.36-159.41)	202.79(80.7-410.20)	69.39(27.84-142.56)	−0.29(−0.33 to −0.25)
Eastern Sub-Saharan Africa	134.87(53.66-274.53)	119.47(47.55-242.76)	219.74(89.46-465.54)	82.03(33.23-169.00)	−1.57(−1.76 to −1.39)
High-income Asia Pacific	116.84(45.92-253.87)	56.65(22.29-123.57)	178.91(70.63-356.71)	57.83(22.77-117.71)	−0.09(−0.40 to 0.22)
High-income North America	210.37(83.44-448.35)	66.30(26.35-142.02)	316.57(126.39-636.54)	61.88(24.94-126.08)	−0.47(−0.89 to −0.04)
North Africa and Middle East	139.42(54.46-305.55)	62.44(24.47-135.68)	448.89(180.27-924.57)	75.15(30.20-154.11)	0.71(0.61 to 0.81)
Oceania	2.63(1.03-5.79)	64.39(25.36-138.53)	1.45(0.55-3.20)	14.37(5.36-31.85)	−6.66(−7.64 to −5.66)
South Asia	852.78(346.10−1,746.63)	105.20(42.21-215.03)	2,028.69(829.37−4,143.64)	113.39(46.48-230.17)	0.16(0.10 to 0.22)
Southeast Asia	240.37(95.32-521.97)	71.35(28.10-154.11)	485.82(195.24-971.33)	65.05(26.09-130.61)	−0.49(−0.60 to −0.39)
Southern Latin America	37.49(14.77-81.00)	79.65(31.39-172.16)	65.89(26.11-135.37)	84.17(33.02-173.78)	0.19(0.03 to 0.36)
Southern Sub-Saharan Africa	15.50(5.99-33.90)	46.32(17.87-101.39)	25.05(9.86-52.96)	35.83(13.80-75.14)	−1.05(−1.24 to −0.86)
Tropical Latin America	83.89(33.55-179.74)	69.96(27.96-150.81)	210.54(85.87-438.94)	80.01(32.66-167.02)	0.47(0.16 to 0.79)
Western Europe	330.19(129.98-679.81)	70.07(27.86-145.68)	384.37(150.46-787.25)	61.33(24.15-127.33)	−0.62(−0.83 to −0.41)
Western Sub-Saharan Africa	183.73(73.84-365.92)	141.78(57.31-283.61)	282.18(113.97-589.75)	86.54(34.51-176.53)	−2.02(−2.22 to −1.81)

**Note:** UI: Uncertainty interval, CI: Confidence interval, EAPC: Estimated annual percent change, SDI: Socio-demographic index, YLDs: Years Lived with Disability.

**Fig 1:**
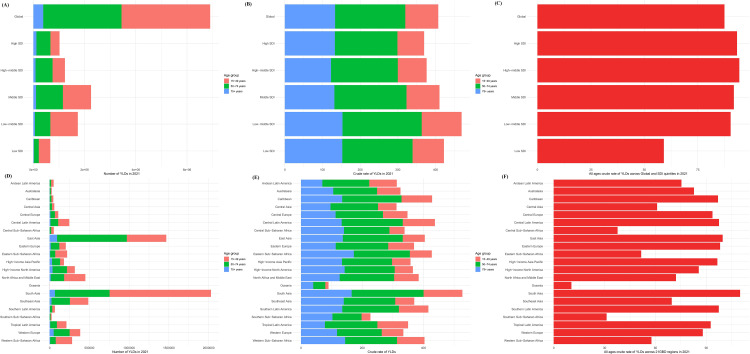
The number and crude rate of YLDs for the age groups 15-49, 50-74, 75 + years and all ages in the global, SDI quintiles, and 21 GBD regions in 2021. (A) The number of YLDs for the age groups 15-49, 50-74, 75 + years and all ages in the global and SDI quintiles regions in 2021, (B) The crude rate of YLDs for the age groups 15-49, 50-74, and 75 + years in the global and SDI quintiles regions in 2021, (C) The crude rate of YLDs for all ages in the global and SDI quintiles regions in 2021, (D) The number of YLDs for the age groups 15-49, 50-74, 75 + years and all ages in 21 GBD regions in 2021, (E) The crude rate of YLDs for the age groups 15-49, 50-74, and 75 + years in 21 GBD regions in 2021, (F) The crude rate of YLDs for all ages in 21 GBD regions in 2021.

The Number of YLDs varied significantly across SDI quintiles in 2021, ranked as follows: Middle SDI > Low-Middle SDI > High-Middle SDI > High SDI > Low SDI (Fig 1A). The highest proportion of YLDs number in the Global, Middle SDI, Low-Middle SDI, and Low SDI categories is found in the 15–49 years age group, followed by the 50–74 years group, and then the 75 + years group. However, in the High SDI and High-Middle SDI categories, the highest proportion of YLDs number is found in the 50–74 years age group (Fig 1A). The crude rate of YLDs in 2021 across Global and the SDI quintiles regions, from highest to lowest, is as follows: 50–74 years, 75 + years, and 15–49 years (Fig 1B). The crude rate of YLDs varied significantly across SDI quintiles in 2021, ranked as follows: High-Middle SDI > High SDI > Middle SDI > Low-Middle SDI > Low SDI (Fig 1C). In the 21 GBD regions, South Asia has the highest number of YLDs, with the greatest number of YLDs in the 15–49 years age group (Fig 1D). Additionally, the crude rate of YLDs is highest in the 50–74 years age group, as well as for all ages (Fig 1E, 1F).

### SDI region level

**Fig 2:**
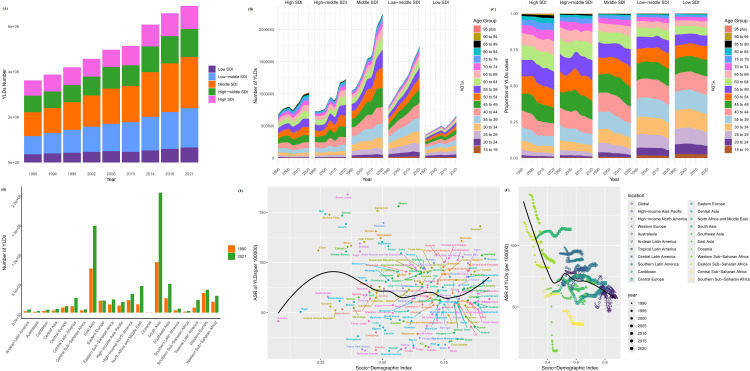
The YLDs of severe periodontitis. (A) All SDI quintiles showed an increasing trend in the number of YLDs; (B) The number of YLDs in different age groups of all SDI quintiles; (C) The proportion of YLDs in different age groups of all SDI quintiles; (D) ASR of YLDs for global and all SDI quintiles from 1990 to 2021; (E) Association between ASR of YLDs and SDI in 204 countries and territories; (F) Association between ASR of YLDs and SDI in global and 21 GBD regions. ASR, age-standardized rate; SDI, socio-demographic index; YLDs, years lived with disability.

From 1990 to 2021, all SDI quintiles exhibited significant increases in the number of YLDs, with the largest absolute rise observed in the Middle SDI region (Table 1, Fig 2A-B). Within the Low SDI region, the 35–39 age group accounted for the highest proportion of YLDs. In contrast, as SDI increased, the burden shifted to older age groups, with the 55–59 age group dominating in the High SDI region (Fig 2C).

The ASR of YLDs varied significantly across SDI quintiles in 2021, ranked as follows: Low-Middle SDI > Low SDI > Middle SDI > High-Middle SDI > High SDI (Table 1). Geographically, all 21 regions experienced an increase in YLDs, with South Asia and East Asia reporting the largest absolute growth (Fig 2D). In 2021, South Asia recorded the highest ASR of YLDs, while Oceania reported the lowest (Table 1). Temporal trends in the ASR of YLDs varied across regions, with Australasia exhibiting the fastest increase (EAPC: 0.93%, 95% CI: 0.75–1.12) (Table 1). This positive EAPC reflects a consistent upward trend in the age-standardized burden, indicating a worsening situation in this region. At the national level, the ASR of YLDs increases with SDI when it is below 0.31 or above 0.75, while it decreases as SDI increases between 0.31 and 0.75 (Fig 2E). A significant negative correlation was observed between SDI and ASR of YLDs at regional levels (ρ = −0.49, P < 0.001), indicating that higher SDI regions generally experienced lower ASR of YLDs (Fig 2F).

In 2021, India had the highest number of YLDs (1,540.19 thousand, 95% UI: 633.00–3120.73 thousand), followed by China (1428.45 thousand, 95% UI: 563.79–2917.32 thousand) (S1 Table). The highest ASR of YLDs was reported in Sierra Leone (167.83 per 100,000 population, 95% UI: 65.81–339.06), followed by Gambia (163.33 per 100,000 population, 95% UI: 65.18–327.93). Between 1990 and 2021, Turkey experienced the fastest increase in ASR of YLDs globally, with an EAPC of 2.55% (95% CI: 1.97–3.14), followed by Sierra Leone (EAPC: 2.04%, 95% CI: 1.17–2.91) (S1 Table). These relatively high positive EAPC values indicate a rapid and sustained escalation in the age-standardized burden in both countries.

### Decomposition analysis of severe periodontitis -related YLDs

**Fig 3:**
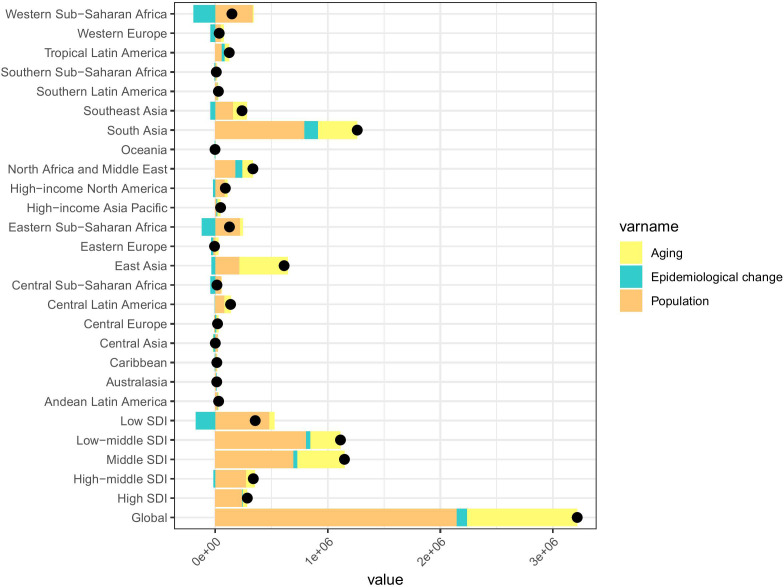
Changes in severe periodontitis YLDs according to population-level determinants of population growth, aging, and epidemiological change from 1990 to 2021 at the global level, 21 GBD regions and by SDI quintile. The black dot represents the overall value of change contributed by all 3 components. For each component, the magnitude of a positive value indicates a corresponding increase in severe periodontitis YLDs attributed to the component; the magnitude of a negative value indicates a corresponding decrease in severe periodontitis YLDs attributed to the related component. GBD, Global Burden of Disease; SDI: Socio-demographic index; YLDs, years lived with disability.

From 1990 to 2021, the global increase in YLDs attributable to severe periodontitis was driven primarily by population growth (66.73%), followed by aging (30.50%) and epidemiological changes (2.77%) (Fig 3, S2 Table). The greatest increase in absolute YLDs number occurred in Middle (694487.10 [60.48%]) and Low-Middle SDI (809070.37 [72.70%]) regions, where population growth contributed 60.48% and 72.70% of the increase, respectively. Compared with 1990, the fastest increase rate in the number of YLDs was observed in the Low SDI, with a 134.48% increase in YLDs attributable to Population growth (Fig 3, S2 Table).

At the regional level, most GBD regions experienced an increase in YLDs attributed to severe periodontitis over the 32-year period. Notable exceptions included Eastern Europe and Oceania, which reported decreases in YLDs due to the combined effects of aging (−521.15%) and population growth (−155.72%), respectively. South Asia and East Asia saw the largest increases in YLDs, driven primarily by population growth (62.92% in South Asia) and aging (70.31% in East Asia).

### YLDs-based frontier analysis

An inverse relationship was observed between SDI and the ASR of YLDs due to severe periodontitis, with the effective frontier (EF) declining as SDI increased (S3 Table). Beyond an SDI threshold of 0.40, the ASR of YLDs stabilized relative to EF.

Countries with the lowest EFs included Somalia, Kiribati, Solomon Islands, Vanuatu, Micronesia (Federated States of), Papua New Guinea, Marshall Islands, Tuvalu, Samoa, and Spain (range: 0–3.26). In contrast, countries with the highest EFs included Gambia, Cabo Verde, Sierra Leone, Ghana, Cameroon, Pakistan, Guinea, Denmark, Burkina Faso, and Bhutan (range: 119.21–151.82).

### Cross-country inequalities of severe periodontitis, 1990–2021

**Fig 4:**
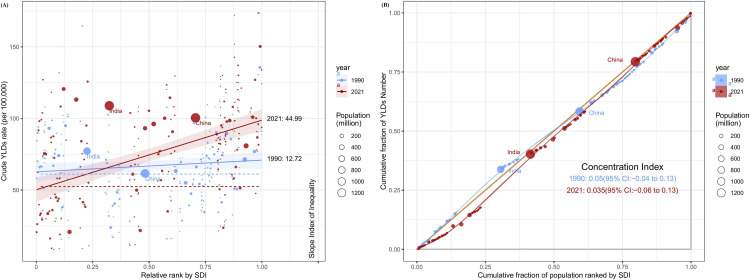
Health inequality regression curves and concentration curves for the YLDs of severe periodontitis worldwide, 1990 and 2021. YLDs, years lived with disability.

Between 1990 and 2021, the Slope Index of Inequality (SII) for YLDs increased from 12.72 (95% CI: 1.92–23.52) to 44.99 (95% CI: 31.14–58.85), reflecting a growing absolute disparity in the crude of YLDs between high-SDI and low-SDI countries (Fig 4A).

The Concentration Index decreased from 0.05 (95% CI: −0.04 to 0.13) in 1990 to 0.035 (95% CI: −0.06 to 0.13) in 2021. Compared with 1990, the CI decreased slightly in 2021, indicating that the health burden of periodontitis has become more evenly distributed among different groups. However, the CI values remain close to zero and the confidence intervals include zero, indicating that the health burden of periodontitis is still not significantly concentrated in a particular socioeconomic group (Fig 4B).

### Future Projections

**Fig 5:**
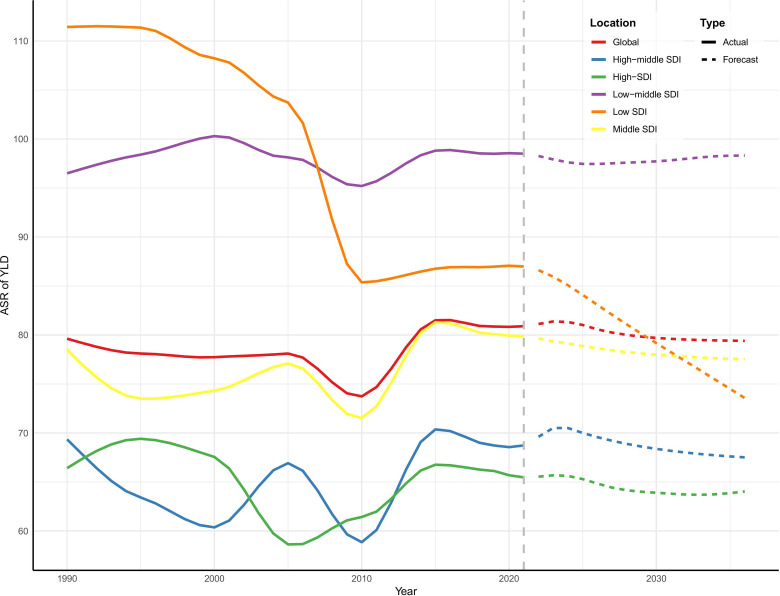
Future forecasts of ASR of YLDs for severe periodontitis at the global level and across five SDI regions over the next 15 years.

Using the ARIMA model fitted to historical ASR of YLDs (1990−2021), we forecasted trends for 2022−2036 at the global level and across the five SDI quintiles. The projections indicate a relatively stable or slightly declining global ASR of YLDs, decreasing from 81.12 in 2022 to 79.40 in 2036 (−2.12%). High-SDI and High-Middle SDI regions are expected to experience modest declines in ASR (−2.31% and −3.02%, respectively), while the Low-Middle SDI region is projected to remain almost unchanged (+0.05%). Notably, the Low-SDI region is forecasted to show the largest reduction in ASR (−15.06%) (Fig 5).

## Discussion

This study provides updated and comprehensive data on YLDs due to severe periodontitis from 1990 to 2021 at the global, regional, and national levels. It includes analyses of the correlation between disease burden and the SDI, as well as decomposition, frontier, and inequality analyses. A significant negative correlation between SDI and the ASR of YLDs was observed at the regional level. The decomposition analysis revealed that population growth and aging are the primary contributors to the increasing global burden of severe periodontitis. The frontier analysis highlighted that low-income countries are substantially distant from the frontier line, indicating considerable potential for reducing the burden. The inequality analysis demonstrated widening disparities between high-income and low-income countries, although relative inequality among high-income countries has narrowed. The ARIMA model projected a continued stable trend in the global ASR of YLDs over the next 15 years if current conditions persist.

Similar to the findings of Nascimento *et al.* [15], our study found that the global number of YLDs due to severe periodontitis increased by 90.67% between 1990 and 2021, reaching 6,903.28 thousand YLDs (95% UI: 2,772.28–14,106.18 thousand) in 2021. Despite this substantial increase, the ASR of YLDs showed only a slight rise, maintaining a stable trend during the same period. The global burden of periodontitis was concentrated primarily in the 50–59 year age group, consistent with the findings of Zhang *et al.* [25]. Although the crude rate of YLDs for the 50–74 year age group is highest globally and across the SDI quintiles, the largest number of YLDs in low-income countries is concentrated in the 15–49 year age group. This pattern is likely due to the shorter life expectancy in these regions compared with the global average. [26].

This study found that the crude rate of YLDs was highest in High-Middle SDI and High SDI regions and lowest in Low SDI regions, whereas the ASR of YLDs was lowest in High SDI regions and highest in Low SDI regions. This pattern may be explained by the fact that high-income countries typically have more robust healthcare resources and better public health systems, leading to more cases being identified and recorded [27]. In contrast, low-income countries often face challenges such as limited healthcare resources, clinical personnel shortages, limited research infrastructure and inadequate health management, resulting in delayed diagnosis and treatment, as well as lower diagnostic rates [28]. Additionally, the younger population structure in these regions-with a higher proportion of younger individuals-contributes to a higher ASR of YLDs.

The South Asia and East Asia regions experienced the greatest growth in the number of YLDs, driven largely by the substantial populations of India and China, which recorded the highest numbers of YLDs in 2021. In South Asia, which falls within the Low-Middle SDI category, the ASR of YLDs was the highest among all regions. The widespread practices of betel nut chewing and tobacco use in this region are likely major contributors to the burden of periodontitis [29].

Australasia exhibited the fastest growth in the ASR of YLDs among all 21 GBD regions, consistent with trends in the 2019 GBD database reported by Zhang *et al*. [25]. The increasing periodontal disease burden among Australian adults may be partly attributed to greater tooth retention, as evidenced by a decrease in the proportion of edentulous individuals from 6.4% to 4% and a reduction in the average number of teeth lost due to pathology from 4.6 to 4.4 [30]. Older individuals who retain more teeth are at a higher risk of developing periodontitis, underscoring the age-related nature of the disease.

This study found a significant negative correlation between ASR of YLDs due to severe periodontitis and SDI at the regional level from 1990 to 2021, continuing the trend of negative correlation observed from 1990 to 2019 [3,25]. High-SDI regions benefit from stronger economic resources and more developed healthcare systems, resulting to lower ASR of YLDs. High-income countries allocate approximately 5–10% of their annual public health budgets to dental care, whereas most low-income countries lack dedicated funding for oral health programs [31].

In 2021, Sierra Leone reported the highest ASR of YLDs globally, and along with Turkey, exhibited the fastest growth rates in disease burden. Sierra Leone’s fragile healthcare system-affected by the civil war (1991–2002)-the Ebola virus outbreak (2013–2015) and recent natural disasters-has limited capacity to address non-life-threatening conditions such as periodontitis [32]. In Turkey, the high prevalence of tobacco use, with nearly half of males aged 15 and older being smokers, is a major factor contributing to the rapid increase in periodontal disease burden [33,34].

Population growth and aging emerged as the most significant contributors to the increasing global burden of periodontitis-related YLDs, consistent with findings by Chen *et al*. [3]. While Chen *et al*.’s analysis was limited to global-level trends, this study provides detailed decomposition analyses across SDI quintiles and 21 GBD regions. These analyses offer a comprehensive understanding of the impact of population growth, aging, and epidemiological changes on the burden of periodontitis in regions and countries with varying economic levels. Proactively implementing preventive strategies in response to population growth and aging trends is crucial for mitigating the periodontal disease burden.

Several studies have utilized the GBD database to illustrate the burden of periodontal disease at the global, regional, and national levels [3,15,25,35,36], but none have deeply explored the potential for improvement in this burden. Our frontier analysis showed that many countries remain far from the frontier line, indicating considerable potential for reducing periodontitis-related YLDs. Interestingly, Denmark, a high-SDI country with an advanced healthcare system, ranked among the top 10 countries with the highest EFs. This suggests shortcomings in its public health policies for periodontal disease prevention, possibly due to the lack of direct monitoring of dental care quality and the reliance on out-of-pocket expenses for adults dental treatment [37].

Quantifying cross-country inequalities in the burden of severe periodontitis across the SDI gradient can provide insights into burden distribution patterns and help identify countries where improvements in the prevention and control of severe periodontitis are most needed [38]. High-SDI countries bear a disproportionately higher burden of periodontitis, possibly due to increased tooth retention and associated periodontal risks. Li *et al*. [39] observed that from 1990 to 2019, High-SDI and High-Middle SDI regions experienced a decline in the ASR of incidence, prevalence, and disability-adjusted life-years (DALYs) for edentulism, suggesting increased tooth retention and a higher risk of periodontitis. These countries have better access to healthcare, which results in a higher number of diagnosed cases, and they retain more teeth in older populations, thereby increasing the risk of developing severe periodontitis. Consequently, the crude rate of YLDs in high-SDI countries is higher. In contrast, Low-SDI and Low-Middle SDI regions showed rising prevalence and DALY rates for edentulism, indicating fewer retained teeth and a reduced periodontitis burden. In these regions, financial and resource constraints often make tooth extraction the most economical option, further limiting access to dental care [40].

The observed increase in the SII and the slight reduction in the Concentration Index suggests that, while absolute health disparities have grown over time, the relative concentration of health outcomes across socio-economic groups has remained relatively stable. This may reflect a situation in which interventions aimed at improving health in lower socio-economic groups have had some effect, but not enough to substantially reduce the overall health inequality gap, resulting in increased absolute disparities while maintaining relatively stable relative inequalities.

To address these disparities, country-specific strategies should focus on equitable healthcare resource allocation. In high-SDI countries, preventive oral health policies and integration of periodontal care within broader healthcare frameworks are essential. In Low-SDI countries, improving access to basic oral health services and community-based interventions is critical to reducing the burden of periodontitis. These strategies align with the WHO Global Oral Health Action Plan 2023–2030, which prioritizes integrating oral health into universal health coverage and primary health care [41]. Strengthening workforce capacity, improving oral health literacy, and establishing sustainable financing mechanisms are critical to achieving equitable outcomes. International partnerships and monitoring frameworks should be leveraged to ensure accountability and track progress toward global targets.

The ARIMA model forecasts suggest that, over the next 15 years, the global ASR of YLDs for severe periodontitis will remain relatively stable or show a slight decline, with notable heterogeneity across SDI regions. High- and High-Middle SDI regions are projected to experience modest decreases, highlighting the importance of maintaining current preventive policies, integrating periodontal care into primary healthcare, and addressing the needs of aging populations with retained dentition. In contrast, the Low-Middle SDI region is expected to remain essentially unchanged, indicating the need for improved access to basic oral healthcare and community-based interventions. The most substantial decline is forecasted in the Low-SDI region, yet the baseline burden remains high; thus, resource allocation should prioritize cost-effective preventive programs, public awareness campaigns, and workforce strengthening to ensure sustainable reductions in disease burden.

This study has several limitations. First, the GBD 2021 data rely on modelled estimates, which may not fully capture country-specific variations in data quality and reporting accuracy. Second, the case definitions of severe periodontitis used in the GBD study may differ from clinical definitions applied in various regions, potentially affecting the comparability of results. Third, this analysis does not consider individual-level risk factors such as smoking, oral hygiene practices, or access to dental care, which are key determinants of periodontitis burden. Moreover, the reliance on the CPITN in the GBD case definition may underestimate the prevalence and severity of periodontitis compared with more robust case definitions, leading to lower estimates than those reported in other epidemiological studies [42]. Finally, using the socio-demographic index (SDI) as a proxy for development may oversimplify the complex interactions between socioeconomic factors and oral health disparities.

## Conclusion

In conclusion, the findings of this study highlight the growing disparities in the burden of severe periodontitis across countries with differing socio-demographic indices, underscoring the urgent need for targeted oral health policies and interventions. Between 1990 and 2021, the global number of YLDs due to periodontitis increased substantially, driven primarily by population growth and aging. While the global ASR of YLDs remained relatively stable, significant cross-country disparities have widened, with higher SDI countries shouldering a disproportionately greater burden. These findings emphasize the need for targeted interventions and policies to reduce inequalities and improve oral health outcomes across all countries, regardless of their socio-demographic development levels. Moreover, they underscore the importance of integrating oral health into broader global health agendas to ensure it is prioritized within international monitoring frameworks.

## Supporting information

S1 Table YLDs of severe periodontitis in 1990 and 2021 for both sexes and all locations, with EAPC from 1990 to 2021. EAPC, estimated annual percentage changes; YLDs, years lived with disability.
(DOCX)

S2 Table Decomposition analysis of change in YLDs due to severe periodontitis in global, all SDI quintiles and 21 GBD regions. GBD, Global Burden of Disease; SDI: Socio-demographic index; YLDs, years lived with disability.
(DOCX)

S3 Table Frontier analysis involving SDI and periodontitis-related ASR of YLDs in 2021. (DOCX)

S1 File Statement. (DOCX)
