# Supplementary material for: Temporal trends and health inequalities in global, regional, and national years lived with disability of severe periodontitis from 1990 to 2021
Source: PLoS One. 2026 Feb 2;21(2):e0337994. doi: 10.1371/journal.pone.0337994 (PMC12863517; doi:10.1371/journal.pone.0337994)
Supplement: S1 Table — EAPC, estimated annual percentage changes; YLDs, years lived with disability. (DOCX) [file pone.0337994.s001.docx]

**Supplementary Table S1**. YLDs of severe periodontitis in 1990 and 2021 for both sexes and all locations, with EAPC from 1990 to 2021.

| **Location** | 1990 | | 2021 | | EAPC % (95% CI) 1990-2021 |
| --- | --- | --- | --- | --- | --- |
|  | YLDs Number (95% UI) | Age-standardized incidence per 100 000 population (95% UI) | YLDs Number (95% UI) | Age-standardized incidence per 100 000 population (95% UI) |  |
| **Nation** |  |  |  |  |  |
| Afghanistan | 3546.06(1367.05-7862.98) | 49.25(18.93-108.88) | 9179.3(3606.68-20041.11) | 53.24(21.17-112.67) | 0.49(0.40 to 0.58) |
| Albania | 1248.94(481.15-2755.25) | 49.01(18.98-107.46) | 2063(802.95-4243.36) | 57.31(22.61-118.78) | 0.69(0.61 to 0.76) |
| Algeria | 10779.6(4247.05-23832.87) | 68.98(27.5-151.9) | 33154.35(13125.5-68525.28) | 75.57(30.02-155.41) | 0.26(0.23 to 0.29) |
| American Samoa | 27.37(11.05-57.25) | 85.5(34.24-179.7) | 42.7(17.2-90.7) | 81.18(32.66-171.44) | -0.11(-0.12 to -0.09) |
| Angola | 7631.1(3055.33-15440.58) | 117.53(47.29-239.77) | 14377.1(5718.63-30086.66) | 76.58(30.73-159.14) | -1.9(-2.28 to -1.52) |
| Antigua and Barbuda | 58.89(23.32-119.08) | 110.1(44.15-223.91) | 109.43(43.05-226.19) | 100.18(39.74-209.83) | -0.32(-0.43 to -0.22) |
| Argentina | 24443.15(9607.09-53236.8) | 76.44(30.09-166.45) | 40447.13(15964.18-83189.13) | 78.87(30.95-162.88) | 0.29(0.05 to 0.52) |
| Armenia | 1960.63(788.01-4289.02) | 63.75(25.5-139.89) | 2305.11(923.79-4706.22) | 60.11(24.05-123.84) | -0.15(-0.35 to 0.05) |
| Australia | 9687.87(3731.38-21349.45) | 52.18(20.18-114.82) | 20155.7(7979.84-42739.52) | 60.26(23.18-129.19) | 0.99(0.78 to 1.2) |
| Austria | 6186.7(2422.43-13638.76) | 64.75(25.48-143.27) | 7706.24(2981.56-16153.91) | 58.74(22.98-125.13) | -0.35(-0.57 to -0.13) |
| Azerbaijan | 3950.18(1579.88-8584.27) | 68.45(27.38-148.91) | 7922.5(3151.19-16611.13) | 64.71(25.5-135.11) | -0.09(-0.38 to 0.2) |
| Bahamas | 246.61(97.73-498.32) | 114.54(46.04-233.39) | 457.48(180.7-955.72) | 102.32(40.29-213.91) | -0.29(-0.34 to -0.23) |
| Bahrain | 314.73(125.09-677.87) | 81.3(33.04-174.53) | 1561.94(613.53-3310.1) | 88.17(34.88-183.07) | 0.3(0.25 to 0.35) |
| Bangladesh | 80778.67(32103.22-166472.77) | 116.37(45.93-236.71) | 198443.57(77177.36-403078.2) | 124.34(48.72-252.2) | 0.18(0.16 to 0.19) |
| Barbados | 273.9(110.11-558.2) | 108.11(43.35-220.77) | 380.7(149.99-769.44) | 95.49(37.42-196.5) | -0.47(-0.55 to -0.4) |
| Belarus | 8655.89(3462.13-18862.58) | 70.85(28.19-154.25) | 9378.74(3758.87-18933.08) | 69.39(27.7-143.1) | 0.03(-0.12 to 0.18) |
| Belgium | 12468.98(5005.81-25698.79) | 102.5(40.95-212.75) | 14909.61(5955.63-30389.54) | 97.67(38.91-204.08) | -0.32(-0.49 to -0.14) |
| Belize | 109.39(43.43-230.33) | 94.77(37.55-197.35) | 344.9(134.99-736.83) | 88.68(35.49-187.16) | -0.3(-0.4 to -0.21) |
| Benin | 4383.01(1741.57-8739.89) | 146.44(58.74-292.64) | 10163.54(4002.48-21509.29) | 116.67(46.61-243.11) | -0.89(-1.14 to -0.63) |
| Bermuda | 81.02(31.95-163.55) | 117.63(46.73-238.72) | 109.26(44.36-218.32) | 121.43(48.49-243.03) | 0.12(0.11 to 0.14) |
| Bhutan | 487.1(191.85-984.04) | 119.66(47.66-244.17) | 997.33(387.13-1998.51) | 131.13(50.95-259.11) | 0.24(0.21 to 0.27) |
| Bolivia (Plurinational State of) | 3209.76(1296.31-6835.76) | 73.01(29.44-155.14) | 7901.04(3078.66-16774.41) | 70.83(27.43-148.07) | -0.09(-0.22 to 0.04) |
| Bosnia and Herzegovina | 1935.26(749.07-4332.15) | 41.06(15.9-90.24) | 2796.61(1093.63-5837.42) | 57.1(22.56-119.78) | 1.3(1.14 to 1.46) |
| Botswana | 269.52(103.7-591.02) | 36.75(14.03-79.75) | 589.12(218.86-1335.78) | 28.69(10.85-63.32) | -1.35(-1.63 to -1.07) |
| Brazil | 81688.06(32664.37-174988.93) | 69.72(27.88-150.31) | 205231.99(83738.48-428791.7) | 80.08(32.71-167.56) | 0.49(0.17 to 0.81) |
| Brunei Darussalam | 115.07(45.58-253.1) | 70.04(27.88-147.64) | 274.89(108.69-565.16) | 58.38(22.99-117.38) | -0.54(-0.66 to -0.43) |
| Bulgaria | 5115.87(1942.19-11011.48) | 45.04(17.27-97.35) | 5042.1(1927.25-10671.31) | 47.13(18.13-99.68) | 0.35(0.11 to 0.59) |
| Burkina Faso | 8580.72(3392.54-17090.09) | 144.18(57.61-288.2) | 22323.83(8835.16-44720.19) | 147.69(59.41-293.79) | 0.06(0.05 to 0.08) |
| Burundi | 4116.69(1665.01-8442.99) | 121.35(48.75-247.1) | 4282.05(1632.29-9618.69) | 56.09(21.44-121.2) | -3.35(-3.94 to -2.76) |
| Cabo Verde | 361.42(144.02-726.18) | 151.2(60.88-301.19) | 920.19(360.22-1853.33) | 160.37(63.21-320.55) | 0.2(0.17 to 0.22) |
| Cambodia | 3118.15(1215.37-6899.36) | 52.25(20.4-115.05) | 9881.56(3822.32-21568.74) | 64.82(24.98-141.4) | 0.8(0.73 to 0.87) |
| Cameroon | 10569.21(4218.78-21173.48) | 153.02(61.7-302.7) | 34407.57(13629.93-69891.8) | 151.4(60.73-305.26) | -0.03(-0.04 to -0.02) |
| Canada | 29189.45(11845.43-60986.48) | 93.46(37.99-195.82) | 47561.62(18669.62-95608.46) | 90.99(36.06-185.13) | 0.06(0.01 to 0.12) |
| Central African Republic | 1805.37(720.34-3782.43) | 103.13(40.7-212.58) | 1808.09(689.3-3995.65) | 52.36(20.35-113.3) | -2.96(-3.46 to -2.46) |
| Chad | 5399.05(2131.78-10718.57) | 141.02(56.69-281.35) | 11086.51(4305.64-23663.59) | 112(43.97-235.56) | -0.86(-1.11 to -0.61) |
| Chile | 9608.09(3765.78-20594.17) | 81.63(31.78-174.47) | 21023.42(8289.47-43308.95) | 92.2(36.25-191.05) | 0.06(-0.14 to 0.26) |
| China | 724759.75(287789.45-1563000.52) | 72.13(28.83-156.29) | 1428453.47(563790.77-2917316.55) | 70.15(27.97-144.15) | 0.44(0.06 to 0.82) |
| Colombia | 22634.91(8980.81-47330.16) | 92.85(37.17-193.93) | 51677.25(20550.7-107660.76) | 94.82(37.75-197.99) | 0.12(0.06 to 0.17) |
| Comoros | 379.73(147.03-760.18) | 131.76(52.36-259.75) | 436.17(170.29-923.71) | 69.88(27.29-147.1) | -2.82(-3.33 to -2.3) |
| Cook Islands | 11.88(4.79-25.19) | 80.29(32.59-171.32) | 4.99(1.84-10.97) | 21.56(8.07-47.45) | -4.83(-6.2 to -3.44) |
| Costa Rica | 2120.01(841.03-4411.86) | 93.28(37.53-195.16) | 5131.44(2032.32-10583.94) | 94.63(37.44-195.44) | 0.06(0.01 to 0.11) |
| Croatia | 4234.19(1702.85-8689.28) | 70.56(28.47-145.85) | 5064.06(2006.78-10331.48) | 82.45(32.89-170.66) | 0.49(0.39 to 0.59) |
| Cuba | 10918.19(4373.48-22420.35) | 101.42(40.75-209.44) | 13906.87(5614.38-28525.98) | 90.12(36.39-187.38) | -0.19(-0.35 to -0.04) |
| Cyprus | 526.96(202.66-1181.05) | 64.69(25.09-144.69) | 1049.88(403.5-2206.01) | 57.13(21.89-120.35) | -0.48(-0.68 to -0.28) |
| Czechia | 7850.55(3067.66-16895.14) | 63.18(25.01-135.6) | 10459.34(4170.1-20967.71) | 66.46(26.11-135.74) | 0.18(0.13 to 0.22) |
| C么te d'Ivoire | 12035.86(4870.84-24289.59) | 150.45(61.28-304.01) | 24382.53(9529.94-51276.75) | 120.57(47.42-246.54) | -0.9(-1.12 to -0.68) |
| Democratic People's Republic of Korea | 8989.81(3413.28-19247.34) | 47.88(18.25-101.14) | 14543.19(5578.7-31264.2) | 42.59(16.61-90.73) | -0.37(-0.41 to -0.32) |
| Democratic Republic of the Congo | 25550.36(10205.54-52493.7) | 108.93(43.8-223.45) | 30214.59(11578.09-67458.18) | 54.6(20.81-119.03) | -3.02(-3.5 to -2.54) |
| Democratic Sao Tome and Principe | 118.27(47.36-238.68) | 150.08(60.68-300.1) | 212.18(82.17-444.81) | 121.59(47.73-251.24) | -0.63(-0.82 to -0.44) |
| Denmark | 8921.73(3618.45-17980.8) | 143.88(58.2-290.89) | 10161.38(3975.13-19996.2) | 136.17(53.79-272.21) | -0.14(-0.23 to -0.06) |
| Djibouti | 368.17(143.77-749) | 136.87(54.89-272.91) | 780.53(301.39-1639.91) | 75.78(29.25-159.02) | -2.67(-3.16 to -2.17) |
| Dominica | 57.58(23.38-119.01) | 99.06(40.14-205.95) | 72.33(28.8-149.86) | 92.12(36.53-192.98) | -0.28(-0.4 to -0.17) |
| Dominican Republic | 4788.9(1910.98-10137.45) | 94.21(37.65-198.04) | 10349.73(4109.82-21569.23) | 93.71(37.26-194.79) | -0.06(-0.19 to 0.07) |
| Ecuador | 5723.62(2341.29-11761.93) | 79.85(32.05-162.29) | 13858.58(5493.95-29549.83) | 78.32(30.95-166.53) | 0.18(0.07 to 0.28) |
| Egypt | 22375.02(8616.92-49902.53) | 59.16(22.64-129.36) | 64505.17(25271.62-136430.55) | 72.98(28.62-152.51) | 0.72(0.65 to 0.8) |
| El Salvador | 2962.83(1167.5-6385.9) | 82.68(32.44-180.06) | 5325.26(2104.32-11091.2) | 86.17(34.13-179.33) | 0.23(0.21 to 0.26) |
| Equatorial Guinea | 292.05(116.43-604.13) | 108.98(43.34-223.11) | 849.16(335.09-1846.08) | 90.43(35.68-187.85) | -0.86(-1.27 to -0.45) |
| Eritrea | 2463.16(980.66-5077.57) | 120.58(48.24-244.78) | 2923.8(1140.15-6410.52) | 65.22(25.91-138.45) | -2.28(-2.84 to -1.71) |
| Estonia | 1352.84(540.91-2883.34) | 71.36(28.61-153.13) | 1406.81(563.3-2814.19) | 74.16(29.51-150.57) | 0.14(0.1 to 0.17) |
| Eswatini | 146.27(56.81-325.51) | 36.3(13.96-80.06) | 326.35(127.51-722.71) | 41.38(16.04-89.56) | 0.45(0.42 to 0.48) |
| Ethiopia | 33376.77(13447.34-68381.52) | 112.48(45.35-228.84) | 82156.96(33052.29-167433.19) | 115.52(46.7-233.38) | 0.16(0.13 to 0.2) |
| Fiji | 368.32(145.73-793.49) | 70.55(27.76-152.11) | 146.97(54.32-321.78) | 16.6(6.21-36.41) | -6.52(-7.47 to -5.55) |
| Finland | 5235.24(2074.84-11156.44) | 85.45(34.19-180.83) | 6705.81(2644.5-13621.76) | 88.01(34.56-180.29) | 0.02(-0.18 to 0.21) |
| France | 29548.58(11583.51-60816.39) | 42.67(16.81-87.63) | 43886.1(16626.51-95000.26) | 45.95(17.49-97.26) | 0.89(0.63 to 1.15) |
| Gabon | 938.28(368.99-1862.83) | 133.49(53.02-265.82) | 1226.99(479.61-2625.19) | 86.35(33.98-180.64) | -1.97(-2.33 to -1.61) |
| Gambia | 1059.28(405-2152.85) | 165.43(65.91-330.28) | 2830.2(1095.27-5750.5) | 163.66(65.18-327.93) | -0.17(-0.34 to 0) |
| Georgia | 4242.92(1699.89-9162.79) | 69.67(27.81-151.21) | 2975.37(1194.02-6025.02) | 62.09(24.9-127.42) | -0.26(-0.47 to -0.05) |
| Germany | 108374.24(43399.92-220172.95) | 106.6(42.82-215.78) | 128271.08(51475.64-257838.63) | 107.16(42.5-222.34) | -0.6(-1.03 to -0.17) |
| Ghana | 15370.22(6198.95-30861.97) | 150.04(61.2-300.92) | 43042.21(17089.49-86416.73) | 156.01(63.09-310.36) | 0.12(0.11 to 0.13) |
| Greece | 7433.08(2874.09-16342.43) | 57.63(22.23-128.29) | 8931.67(3498.57-18387.74) | 57.12(22.35-120.27) | 0.23(0.01 to 0.44) |
| Greenland | 36.27(14.43-80.57) | 73.01(29.1-158.25) | 51.11(20.38-105.45) | 72.77(28.85-153.52) | -0.04(-0.08 to 0) |
| Grenada | 62.66(25.38-129.79) | 96.76(39.39-201.69) | 110.59(43.39-231.58) | 94.68(37.08-199.25) | -0.13(-0.23 to -0.04) |
| Guam | 101.16(40.14-209.26) | 91.56(36.7-189.92) | 46.37(17.34-101.7) | 23.68(8.9-51.38) | -5.95(-7.04 to -4.84) |
| Guatemala | 4113.24(1641.39-8814.92) | 83.97(33.46-179.57) | 11248.41(4360.46-23813.23) | 84.25(33.34-176.56) | 0.04(-0.01 to 0.09) |
| Guinea | 6222.44(2514.49-12355.9) | 148.04(59.76-295.52) | 13311.76(5271.99-26796.15) | 146.79(58.97-292.45) | -0.08(-0.1 to -0.05) |
| Guinea-Bissau | 921.93(369.98-1845.11) | 144.63(58.18-284.63) | 1515.7(595.95-3335.01) | 110.99(43.79-234.16) | -0.87(-1.09 to -0.65) |
| Guyana | 495.22(196.06-1030.93) | 90.18(36.02-187.76) | 656.59(256.81-1384.57) | 88.44(34.81-185.61) | -0.17(-0.29 to -0.04) |
| Haiti | 3679.42(1472.77-7819.38) | 83.77(33.12-180.01) | 7474.17(2887.46-15967.66) | 70.1(27.36-146.93) | -0.6(-0.73 to -0.48) |
| Honduras | 2273.61(905.44-4821) | 81.91(32.45-174.23) | 6707.78(2599.68-14050.28) | 80.86(31.54-166.87) | 0(-0.05 to 0.05) |
| Hungary | 3578.09(1366.05-7837.13) | 26.99(10.27-59.09) | 4473.19(1650.79-10136.26) | 29.9(11.28-65.87) | 0.39(0.1 to 0.67) |
| Iceland | 166.7(66.92-342.25) | 63.88(25.57-131.88) | 273.42(105.23-578.32) | 60.02(23.3-128.34) | 0.11(0 to 0.22) |
| India | 658055.18(266415.47-1349120.61) | 100.98(40.72-207.93) | 1540186.62(632989.83-3120731.91) | 109.07(44.92-219.72) | 0.15(0.06 to 0.23) |
| Indonesia | 117028.22(46704.41-245614.84) | 85.96(34.12-180.89) | 268116.92(106780.44-566039.17) | 88.94(35.44-187.06) | 0.1(0.08 to 0.12) |
| Iran (Islamic Republic of) | 27658.12(10933.77-58312.96) | 79.55(31.71-168.27) | 77417.79(31278.41-162816.99) | 80.29(32.35-167.97) | 0.21(0.07 to 0.36) |
| Iraq | 7179.49(2821.82-15727.06) | 67.83(27.14-146.78) | 26113.16(10297.95-55172.24) | 75.7(29.91-157.9) | 0.56(0.49 to 0.62) |
| Ireland | 1175.8(450.7-2602.69) | 32.49(12.59-72.26) | 2353.16(882.69-5075.48) | 35.92(13.71-77.1) | 0.65(0.35 to 0.95) |
| Israel | 2948.27(1145.66-6478.04) | 64.57(25.33-141.66) | 5820.14(2278.11-12483.02) | 56.31(21.92-121.45) | -0.53(-0.76 to -0.3) |
| Italy | 48616.11(18908.18-104393.17) | 65.89(25.79-142.98) | 58506.72(23127.58-117755.66) | 60.67(23.9-125.7) | -0.23(-0.28 to -0.17) |
| Jamaica | 1776.04(708.02-3746.92) | 98.29(39.51-206.08) | 2777.83(1108.82-5854.83) | 89.9(35.89-189.28) | -0.36(-0.47 to -0.26) |
| Japan | 96781.54(38180.61-208944.27) | 58.91(23.25-128.69) | 128797.49(50996.07-254615.46) | 59.78(23.5-121.91) | -0.12(-0.52 to 0.28) |
| Jordan | 1327.09(522.93-2913.01) | 64.75(25.62-140.35) | 7944.06(3085.62-16441.46) | 72.3(28.22-149.02) | 0.5(0.45 to 0.55) |
| Kazakhstan | 9833.49(3899.27-21152.59) | 68.41(27.15-148.58) | 13366.55(5322.03-27667.47) | 66.26(26.43-136.49) | -0.12(-0.27 to 0.04) |
| Kenya | 16470.61(6486.4-32935.25) | 126.88(50.3-253.35) | 42084.45(16508.4-88645.61) | 116.14(45.59-236.06) | -0.21(-0.26 to -0.17) |
| Kiribati | 28.62(11.24-62.82) | 59.56(23.21-130.89) | 11.12(4.15-24.45) | 11.83(4.45-25.64) | -7.09(-8.07 to -6.1) |
| Kuwait | 1177.13(461.04-2482.55) | 87.4(34.51-182.89) | 5264.02(2064.69-11225.34) | 91.65(36.7-188.68) | 0.19(0.12 to 0.25) |
| Kyrgyzstan | 2049.83(816.21-4455.47) | 62.84(24.62-135.53) | 3135.69(1234.76-6691.36) | 51.76(20.46-110.88) | -0.81(-1.1 to -0.53) |
| Lao People's Democratic Republic | 647.64(248.82-1413.59) | 25.68(9.56-56.6) | 2210.51(844.59-4889.49) | 37.11(14.03-79.71) | 1.62(1.17 to 2.08) |
| Latvia | 2404.24(972.29-5114.38) | 73.32(29.69-156.3) | 2081.8(821.69-4140.55) | 74.18(29.43-151.96) | 0.09(0.03 to 0.15) |
| Lebanon | 1675.66(670.71-3616.95) | 67.26(26.79-145.07) | 4560.44(1816.67-9465.99) | 75.32(29.86-155.39) | 0.47(0.38 to 0.56) |
| Lesotho | 257.2(97.71-568.4) | 26.23(9.91-57.88) | 441.48(170.69-961.4) | 32.26(12.34-70.14) | 0.73(0.71 to 0.75) |
| Liberia | 2395.68(957.45-4819.96) | 144.26(58.23-288.46) | 5668.58(2222.45-11497.88) | 138.37(55.69-276.77) | -0.04(-0.08 to -0.01) |
| Libya | 2107.96(831.27-4482.76) | 81.82(32.38-174.65) | 5392.5(2151.83-11300.4) | 71.41(28.33-147.61) | -0.33(-0.45 to -0.21) |
| Lithuania | 3124.01(1255.43-6653.46) | 73.37(29.58-156.41) | 3075.83(1229.47-6147.41) | 75.06(29.73-153.76) | 0.2(0.07 to 0.34) |
| Luxembourg | 342.39(134.16-745.12) | 71.19(27.96-155.78) | 584.98(229.96-1221.19) | 65.16(25.62-138.42) | -0.33(-0.53 to -0.12) |
| Madagascar | 9317.5(3689.84-18817.75) | 125.89(50.3-254.72) | 4613.6(1749.19-9995.23) | 26.44(9.87-56.82) | -5.49(-7.01 to -3.95) |
| Malawi | 7077.47(2783.02-14177.86) | 119.91(47.81-244.32) | 6854.88(2605.57-15367.44) | 60.61(23.67-131.77) | -3.03(-3.6 to -2.46) |
| Malaysia | 9348.68(3734.81-20433.87) | 72.59(28.79-156.24) | 16074.73(6239.67-34736.35) | 50.36(19.58-107.79) | -1.75(-2.29 to -1.21) |
| Maldives | 87.44(33.85-190.62) | 71.19(27.3-152.5) | 249.6(96.19-543.29) | 49.86(19.1-105.12) | -1.75(-2.18 to -1.32) |
| Mali | 8137.21(3194.1-16444.62) | 142.4(56.55-287.91) | 22104.37(8739.28-43584.42) | 148.28(59.65-295.27) | 0.12(0.09 to 0.14) |
| Malta | 265.01(103.27-582.37) | 62.8(24.57-137.54) | 392.92(153.15-809.04) | 59.34(23.22-124.47) | -0.28(-0.5 to -0.07) |
| Marshall Islands | 14.22(5.6-31.44) | 63.62(25.1-137.41) | 6.26(2.3-13.48) | 13.36(4.94-29.43) | -6.93(-7.93 to -5.93) |
| Mauritania | 2112.99(846.09-4274.51) | 149.59(60.88-300.34) | 3730.27(1461.69-7684.45) | 120.38(47.41-246.03) | -0.84(-1.06 to -0.61) |
| Mauritius | 661.17(257.27-1434.31) | 70.82(27.99-151.98) | 843.46(322.43-1812.36) | 48(18.44-103.21) | -1.74(-2.09 to -1.39) |
| Mexico | 56539.44(22673.25-116878.75) | 95.61(38.76-198.43) | 129451.76(52808.76-261896.69) | 93.9(38.21-189.96) | 0.05(0.01 to 0.09) |
| Micronesia (Federated States of) | 36.72(14.17-81.1) | 62.11(24.16-134.55) | 11.81(4.36-25.43) | 13.12(4.92-28.17) | -6.89(-8.19 to -5.57) |
| Monaco | 40.63(16.27-85.55) | 90.58(36.07-190) | 47.06(18.75-94.13) | 78.95(31.31-163.74) | -0.71(-0.9 to -0.51) |
| Mongolia | 748.26(293.71-1641.3) | 58.52(22.81-127.5) | 1910.53(753.52-4060.99) | 59.99(23.8-125.25) | 0.01(-0.13 to 0.14) |
| Montenegro | 385.39(149.6-841.96) | 59.03(22.83-128.38) | 504.46(196.77-1027.61) | 60.2(23.53-123.64) | 0.23(0.11 to 0.34) |
| Morocco | 17474.32(6954.96-36373.5) | 94.6(37.49-195.7) | 35334.9(14289.55-74295.23) | 90.86(36.72-190.48) | 0.03(-0.09 to 0.14) |
| Mozambique | 9369.65(3714.13-19701.52) | 110.01(43.35-227.38) | 10467.25(3969.04-23164.81) | 61.53(23.96-131.6) | -2.69(-3.26 to -2.11) |
| Myanmar | 15350.83(5852.15-33978.54) | 54.22(20.77-117.47) | 23417.11(8835.5-50769.65) | 43.45(16.58-94.57) | -1.21(-1.61 to -0.8) |
| Namibia | 295.06(111.55-648.27) | 35.76(13.42-78.25) | 760(291.38-1648.83) | 41.62(15.93-89.53) | 0.49(0.23 to 0.75) |
| Nauru | 5.42(2.15-11.53) | 81.35(32.71-173.33) | 1.31(0.49-2.86) | 17.04(6.4-37.88) | -6.42(-7.71 to -5.12) |
| Nepal | 9977.37(3903.78-21968.56) | 76.03(29.49-166.71) | 22539.66(8621.78-47990.74) | 81.96(31.44-172.09) | -0.08(-0.47 to 0.31) |
| Netherlands | 11515.2(4478.23-25610.28) | 65.2(25.42-145.61) | 14039.74(5493.39-29316.04) | 57.54(22.54-121.32) | -0.2(-0.4 to -0.01) |
| New Zealand | 2091.38(802.26-4695.2) | 57.35(22.11-129.05) | 5449.35(2172.66-11300.91) | 85.77(34.01-180.8) | 0.77(0.48 to 1.07) |
| Nicaragua | 1830.35(727.49-3806.4) | 83.48(32.95-175.4) | 5027.73(1998.71-10762.54) | 82.43(32.81-174.18) | 0.13(0.07 to 0.2) |
| Niger | 6788.98(2646.63-13673.64) | 140.43(56.16-281.85) | 6555.69(2516.36-14088.33) | 50.8(19.45-107.46) | -2.85(-3.67 to -2.01) |
| Nigeria | 85483.89(33805.67-172261.47) | 137.47(54.94-273.35) | 47956.44(18307.38-105272.32) | 35.11(13.27-75.76) | -5.57(-6.27 to -4.86) |
| Niue | 1.5(0.6-3.22) | 73.47(29.37-158.18) | 0.36(0.13-0.79) | 17.6(6.59-38.41) | -6.32(-7.55 to -5.08) |
| North Macedonia | 1115.65(427.59-2432.89) | 54.48(20.89-118.77) | 1783.57(697.49-3705.56) | 58.15(22.87-122.13) | 0.26(0.21 to 0.31) |
| Northern Mariana Islands | 33.94(13.45-70.72) | 92.41(37.48-195.86) | 12.38(4.54-27.27) | 21.03(7.84-45.46) | -6.12(-7.08 to -5.14) |
| Norway | 5544.66(2239.43-11180.11) | 111.78(44.76-225.88) | 7255.17(2887.51-14894.55) | 103.76(40.87-215.5) | -0.02(-0.19 to 0.16) |
| Oman | 977.37(375.96-2151.64) | 76.91(30.08-166.85) | 4023.17(1598.1-8550.33) | 85.78(34.05-175.01) | 0.43(0.36 to 0.5) |
| Pakistan | 103485.39(41650-206782.72) | 136.23(55.07-272.3) | 266521.11(104382.3-534095.98) | 139.47(55.19-277.83) | 0.15(0.07 to 0.23) |
| Palau | 9.31(3.66-19.79) | 76.27(30.71-162.92) | 4.47(1.62-9.55) | 17.98(6.61-39.11) | -5.1(-6.53 to -3.64) |
| Palestine | 592.25(231.12-1323.61) | 54.48(21.52-120.76) | 2426.7(972.65-5083.4) | 64.49(25.67-133.85) | 0.44(0.34 to 0.54) |
| Panama | 1727.89(692.44-3578.44) | 92.98(37.69-192.87) | 4382.48(1731.85-9066.33) | 99.57(39.25-206.31) | 0.29(0.23 to 0.35) |
| Papua New Guinea | 1552.07(604.65-3414.93) | 61.73(24.11-132.46) | 971.57(365.42-2154.38) | 13.34(4.96-29.54) | -6.86(-8.21 to -5.49) |
| Paraguay | 2202.92(864.39-4689.03) | 79.73(31.38-168.35) | 5306.47(2108.08-11068.74) | 77.92(31.14-161.54) | -0.19(-0.25 to -0.13) |
| Peru | 11704.14(4810.06-24571.05) | 73.8(29.93-153.94) | 28017.48(10952.76-59608.34) | 75.15(29.43-159.74) | 0.26(0.18 to 0.34) |
| Philippines | 29916.37(11754.28-64662.06) | 72.21(28.64-156.89) | 24637.3(9711.09-51485.57) | 24.88(9.74-51.05) | -4.21(-4.57 to -3.85) |
| Poland | 30585.48(12071.06-65296.46) | 72.21(28.6-154.13) | 41305.2(16449.87-84524) | 77.17(30.8-159.89) | 0.33(0.23 to 0.42) |
| Portugal | 7264.06(2793.65-15679.23) | 60.63(23.2-133.21) | 9046.89(3490.59-18587.12) | 54.88(21.07-114.66) | -0.4(-0.61 to -0.19) |
| Principality of Andorra | 48.23(18.97-103.63) | 77.03(30.37-165.76) | 86.79(33.65-180.8) | 64.67(25.16-137.49) | -0.12(-0.29 to 0.04) |
| Puerto Rico | 4038.86(1611.37-8159.67) | 112.53(44.88-227.83) | 4699.75(1855.64-9586.46) | 105.53(41.4-220.98) | -0.25(-0.32 to -0.17) |
| Qatar | 367.73(143.44-774.93) | 96.15(38.59-202.13) | 3575.04(1410.28-7661.25) | 99.29(38.83-204.8) | 0.27(0.22 to 0.33) |
| Republic of Congo | 1839.88(730.89-3680.27) | 119.56(48.03-241.08) | 3074.45(1202.12-6520.2) | 74.19(29.21-154.8) | -2.15(-2.55 to -1.74) |
| Republic of Korea | 18343.44(7006.53-41049.86) | 48.56(18.68-105.75) | 44719.72(17560.05-91907.14) | 53.48(21.12-111.01) | 0.27(0.2 to 0.33) |
| Republic of Moldova | 2910.93(1177.71-6335.49) | 63.44(25.74-137.68) | 3112.61(1213.85-6463.33) | 59.39(23.17-123.98) | -0.15(-0.36 to 0.06) |
| Romania | 15196.65(5779.9-33178.15) | 56.4(21.77-123.41) | 17749.27(6994.89-35918.64) | 63.08(24.84-131.64) | 0.5(0.38 to 0.62) |
| Russian Federation | 135229.61(54324.09-284208.34) | 77.22(31.02-161.99) | 142732.24(56786.65-290218.65) | 70.92(28.37-146.14) | -0.3(-0.33 to -0.26) |
| Rwanda | 5229.44(2065.85-10466.57) | 121.33(48.4-244.37) | 6227.64(2378.02-13351.87) | 67.62(26.2-144.3) | -2.68(-3.21 to -2.15) |
| Saint Kitts and Nevis | 34.7(13.77-70.65) | 104.87(42.01-211.88) | 75.78(30.33-158.1) | 101.02(40.11-211.96) | -0.24(-0.31 to -0.18) |
| Saint Lucia | 99.43(39.98-206.02) | 100.79(40.79-209.3) | 213.33(84.27-438.62) | 93.66(36.54-194.27) | -0.31(-0.42 to -0.2) |
| Saint Vincent and the Grenadines | 75.14(30.03-154.91) | 96.6(38.97-199.01) | 123.18(49.32-252.06) | 92.37(36.75-190.86) | -0.21(-0.31 to -0.11) |
| Samoa | 67.28(26.27-145.71) | 66.34(26.35-143.45) | 24.27(9.01-52.05) | 14.78(5.51-32.1) | -6.63(-7.88 to -5.35) |
| San Marino | 21.56(8.47-46.6) | 74.63(29.46-161.66) | 31.25(12.22-64.17) | 61.74(24.43-131.46) | -0.64(-0.8 to -0.48) |
| Saudi Arabia | 4557.58(1832.64-9618.9) | 47.31(18.78-101.36) | 21932.05(8428.61-48242.55) | 55.28(21.76-116.47) | 0.21(0.08 to 0.35) |
| Senegal | 7287.59(2938.52-14762.15) | 149.34(60.73-298.84) | 13728.68(5421.3-28411.56) | 118.8(47.11-246.28) | -0.99(-1.16 to -0.82) |
| Serbia | 6566.62(2538.81-14342.88) | 57.2(22.21-125.28) | 7471.03(2895.84-15586.2) | 58.81(22.83-122.56) | 0.24(0.11 to 0.36) |
| Seychelles | 47.32(18.89-100.56) | 80.72(32.19-173.29) | 67.75(25.84-143.75) | 53.44(20.49-112.43) | -2.05(-2.5 to -1.6) |
| Sierra Leone | 3228.71(1259.35-6673.51) | 110.69(42.69-227.53) | 11263.3(4322.07-23082.38) | 167.83(64.81-339.06) | 2.04(1.17 to 2.91) |
| Singapore | 1601.76(628.58-3502.05) | 56.81(22.07-124.17) | 5121.82(1985.42-10313.6) | 59.99(23.28-120.73) | 0.19(0.11 to 0.28) |
| Slovakia | 3334.82(1296.21-7292.49) | 58.21(22.66-127.97) | 5004.62(1965.42-10196.35) | 63.8(25.11-132.13) | 0.39(0.35 to 0.44) |
| Slovenia | 2026.1(819.91-4047.98) | 87.08(35.24-173.57) | 2589.36(1014.96-5368.11) | 85.79(33.64-178.73) | 0(-0.09 to 0.08) |
| Solomon Islands | 105.28(41.54-236.86) | 57.33(22.42-126.21) | 57.75(21.5-123.79) | 12.2(4.52-26.63) | -6.93(-7.94 to -5.91) |
| Somalia | 4609.94(1826.01-9696.78) | 102.14(39.88-215.04) | 4448.76(1684.2-9980.16) | 41.48(15.77-91.04) | -3.86(-4.33 to -3.39) |
| South Africa | 12782.62(4940.38-28106.16) | 50.72(19.55-110.81) | 19965.24(7853.63-43883.85) | 36.78(14.2-80.9) | -1.27(-1.49 to -1.06) |
| South Sudan | 5130.2(1999.23-10302.59) | 138.16(55.79-274.33) | 3844.77(1484.41-8293.42) | 65.89(25.87-139.97) | -2.97(-3.28 to -2.65) |
| Spain | 29051.5(11101.65-63991.3) | 64.02(24.37-143.04) | 10791.28(4066.72-23543.27) | 14.9(5.6-31.82) | -5.34(-6.91 to -3.73) |
| Sri Lanka | 16522.75(6530.26-33496.64) | 113.32(45.16-232.04) | 12898.81(4874.07-27795.28) | 48.37(18.47-104.3) | -3.91(-4.75 to -3.06) |
| Sudan | 6005.34(2344.77-13152.05) | 50.56(19.54-110.32) | 16621.64(6435.99-36474.23) | 57.37(22.33-124.6) | 0.55(0.51 to 0.6) |
| Suriname | 324.04(130.09-669.23) | 103.24(41.45-211.35) | 606.41(238.38-1258.47) | 94.8(37.19-200.23) | -0.25(-0.37 to -0.12) |
| Sweden | 8979.15(3582.02-19234.45) | 80.28(31.66-174.17) | 9975.42(3972.23-20068.09) | 68.92(27.44-140.28) | 0.07(-0.08 to 0.22) |
| Switzerland | 6241.96(2478.06-13382.66) | 71.94(28.37-154.43) | 8362.03(3280.34-17183.69) | 63.55(25.17-132.67) | -0.22(-0.38 to -0.07) |
| Syrian Arab Republic | 4061.63(1605.83-8912.74) | 58.1(22.85-125.52) | 9519.4(3705.34-19842.18) | 63.85(25.15-133.63) | 0.31(0.22 to 0.4) |
| Taiwan (Province of China) | 10936.36(4252.76-22981.62) | 60.46(23.25-127.65) | 23664.01(9139.81-49182.11) | 62.47(24.22-131.82) | 0.06(-0.03 to 0.15) |
| Tajikistan | 1991.84(795.57-4280.21) | 62.27(24.84-133.14) | 4047.58(1586.06-8815.66) | 49.75(19.63-105.63) | -0.68(-1.05 to -0.31) |
| Thailand | 30798.36(12153.67-64871.8) | 66.42(26.59-138.88) | 76313.26(30305.32-156340.9) | 77.3(30.51-159.31) | 0.29(0.14 to 0.43) |
| Timor-Leste | 283.24(108.99-627.62) | 60.83(23.67-132.1) | 393.03(150.41-874.64) | 41.52(15.82-92.41) | -1.71(-2.12 to -1.29) |
| Togo | 3265.85(1278.68-6483.67) | 145.27(58.38-287.86) | 6971.93(2717.41-14936.89) | 110.87(44.39-231.92) | -0.87(-1.08 to -0.66) |
| Tokelau | 0.84(0.33-1.82) | 65.57(25.9-141.4) | 0.22(0.08-0.49) | 15.58(5.78-34.15) | -5.05(-6.58 to -3.49) |
| Tonga | 42.03(16.62-92.15) | 66.08(26.2-145.12) | 12.75(4.72-27.34) | 14.9(5.53-32.15) | -6.67(-7.96 to -5.36) |
| Trinidad and Tobago | 1097.16(437.15-2247.33) | 107.53(42.76-218.58) | 1810.07(718.4-3723.86) | 104.11(41.05-215.28) | 0.24(0.13 to 0.36) |
| Tunisia | 3789.03(1494.6-8335.11) | 62.8(25.04-136.85) | 10370.81(4104.1-21504.06) | 74.98(29.59-154.96) | 0.59(0.47 to 0.72) |
| Turkey | 17715.38(6826.22-39279.21) | 41.22(15.93-90.65) | 82121.42(32790.69-170517.72) | 85.02(33.96-177.42) | 2.55(1.97 to 3.14) |
| Turkmenistan | 1565.6(614.46-3420.11) | 67.08(26.48-145.62) | 3245.3(1273.4-6742.95) | 65.82(25.78-135.37) | -0.06(-0.3 to 0.18) |
| Tuvalu | 4.54(1.78-9.8) | 58.98(22.9-127.19) | 1.52(0.55-3.25) | 13.53(4.98-28.98) | -6.7(-7.68 to -5.7) |
| Uganda | 11518.79(4489.7-23278.22) | 119.11(47.33-243.4) | 15602.77(6016.11-34141.08) | 67.47(26.35-142.94) | -2.63(-3.02 to -2.24) |
| Ukraine | 47544.19(19267.95-100540.27) | 73.48(29.64-154.71) | 41005.33(16295.45-83720.84) | 64.48(25.89-134.66) | -0.46(-0.51 to -0.4) |
| United Arab Emirates | 1476.95(580.66-3089.18) | 92.4(37.06-190.51) | 13196.23(5243.35-27917.43) | 91.68(36.36-189.1) | -0.02(-0.12 to 0.09) |
| United Kingdom | 28997.7(11068.23-62481.12) | 40.37(15.55-89.14) | 34845.11(13163.47-73218.97) | 36.04(13.79-77.91) | -0.01(-0.41 to 0.4) |
| United Republic of Tanzania | 19474.74(7754.57-39356.15) | 125.12(50.37-253.06) | 26228.39(10139.59-56607.24) | 70.94(27.65-148.75) | -2.58(-3.09 to -2.07) |
| United States of America | 181136.73(71444.6-387960.24) | 63.33(25.1-136.35) | 268950.53(105735.83-536120.68) | 58.5(23.31-119.15) | -0.57(-1.07 to -0.06) |
| United States Virgin Islands | 119.73(48.44-244.08) | 114.25(45.95-231.41) | 130.33(51.57-265.58) | 109(42.64-229.48) | -0.23(-0.29 to -0.18) |
| Uruguay | 3439.71(1395.2-7056.81) | 103.57(41.97-212.34) | 4412.68(1760.09-8994.79) | 107.9(42.59-223.8) | 0.17(0.11 to 0.23) |
| Uzbekistan | 8224.13(3205.32-17788.61) | 62.25(24.56-134.2) | 19470.51(7570.57-41319.65) | 58.2(22.73-122.27) | -0.34(-0.57 to -0.11) |
| Vanuatu | 52.79(20.42-116.64) | 60.99(23.37-132.99) | 28.5(10.66-63.02) | 12.63(4.67-27.8) | -6.92(-8.21 to -5.61) |
| Venezuela (Bolivarian Republic of) | 13424.8(5367.14-28013.49) | 97.94(39.46-202.52) | 27633.89(11086.49-57528.99) | 90.91(36.27-188.79) | -0.01(-0.08 to 0.06) |
| Viet Nam | 16212.63(6265.16-35301.17) | 35.69(13.5-77.13) | 50041.62(19277.24-106343.14) | 45.5(17.51-96.72) | 1(0.66 to 1.34) |
| Yemen | 4186.86(1684.81-8834.49) | 60.51(23.93-125.72) | 14253.76(5574.42-31296.07) | 64.06(24.91-134.94) | 0.08(-0.05 to 0.21) |
| Zambia | 5871.57(2308.62-11701.36) | 129.14(51.43-259.57) | 8595.43(3297.48-18649.14) | 73.91(28.29-156.28) | -1.99(-2.55 to -1.43) |
| Zimbabwe | 1752.22(674.59-3776.33) | 32.54(12.33-69.34) | 2963.79(1132.12-6558.91) | 30.31(11.6-66.8) | -0.40(-0.52 to -0.27) |

**Note:** UI, Uncertainty interval; CI, Confidence interval; EAPC, estimated annual percentage changes; SDI Socio-demographic index; YLDs: Years Lived with Disability.
