# Supplementary material for: Temporal trends and health inequalities in global, regional, and national years lived with disability of severe periodontitis from 1990 to 2021
Source: PLoS One. 2026 Feb 2;21(2):e0337994. doi: 10.1371/journal.pone.0337994 (PMC12863517; doi:10.1371/journal.pone.0337994)
Supplement: S2 Table — GBD, Global Burden of Disease; SDI: Socio-demographic index; YLDs, years lived with disability. (DOCX) [file pone.0337994.s002.docx]

**Supplementary Table S2**. Decomposition analysis of change in YLDs due to severe periodontitis in global, all SDI quintiles and 21 GBD regions.

| Location | Aging | Population growth | Epidemiological change |
| --- | --- | --- | --- |
| Global | 980806.00 (30.5) | 2146029.85 (66.73) | 89173.37 (2.77) |
| High SDI | 41433.78 (14.52) | 238961.29 (83.75) | 4927.51 (1.73) |
| High-middle SDI | 80373.83 (23.74) | 272182.4 (80.41) | -14050.85 (-4.15) |
| Middle SDI | 421135.99 (36.67) | 694487.10 (60.48) | 32699.7 (2.85) |
| Low-middle SDI | 270198.43 (24.28) | 809070.37 (72.70) | 33648.56 (3.02) |
| Low SDI | 48878.66 (13.74) | 478227.71 (134.48) | -171480.68 (-48.22) |
| Andean Latin America | 9681.79 (31.80) | 20658.57 (67.86) | 102.57 (0.34) |
| Australasia | 1153.26 (8.40) | 8647.31 (62.96) | 3933.57 (28.64) |
| Caribbean | 7316.38 (49.78) | 11936.13 (81.21) | -4554.52 (-30.99) |
| Central Asia | -7098.35 (-652.80) | 23028.6 (2117.83) | -14842.87 (-1365.02) |
| Central Europe | 19967.5 (94.94) | -8061.83 (-38.33) | 9125.92 (43.39) |
| Central Latin America | 58535.72 (42.82) | 80644.03 (59.00) | -2486.98 (-1.82) |
| Central Sub-Saharan Africa | 4125.77 (26.34) | 52179.05 (333.13) | -40641.46 (-259.47) |
| East Asia | 430357.2 (70.31) | 213670.64 (34.91) | -31905.61 (-5.21) |
| Eastern Europe | 30863.38 (-521.15) | -18942.32 (319.85) | -17843.23 (301.30) |
| Eastern Sub-Saharan Africa | 26883.40 (21.24) | 217921.41 (172.17) | -118230.73 (-93.41) |
| High-income Asia Pacific | 30766.03 (64.07) | 10910.45 (22.72) | 6345.89 (13.21) |
| High-income North America | 25996.07 (28.83) | 83597.05 (92.70) | -19408.10 (-21.52) |
| North Africa and Middle East | 95286.39 (28.45) | 178828.14 (53.38) | 60864.79 (18.17) |
| Oceania | 438.71 (-30.33) | 2252.25 (-155.72) | -4137.3 (286.05) |
| South Asia | 348846.01 (27.64) | 794090.17 (62.92) | 119043.62 (9.43) |
| Southeast Asia | 124728.87 (52.35) | 156025.7 (65.48) | -42476.05 (-17.83) |
| Southern Latin America | 8869.32 (31.14) | 16700.83 (58.63) | 2915.23 (10.23) |
| Southern Sub-Saharan Africa | 6194.49 (62.81) | 9750.76 (98.88) | -6083.57 (-61.69) |
| Tropical Latin America | 43138.11 (34.56) | 60301 (48.31) | 21369.3 (17.12) |
| Western Europe | 31382.29 (87.88) | 46401.92 (129.94) | -42074.01 (-117.82) |
| Western Sub-Saharan Africa | 10649.77 (7.16) | 330954.55 (222.63) | -192948.37 (-129.8) |

Note: GBD, Global Burden of Disease; SDI: Socio-demographic index; YLDs, years lived with disability.
