# Supplementary material for: Temporal trends and health inequalities in global, regional, and national years lived with disability of severe periodontitis from 1990 to 2021
Source: PLoS One. 2026 Feb 2;21(2):e0337994. doi: 10.1371/journal.pone.0337994 (PMC12863517; doi:10.1371/journal.pone.0337994)
Supplement: S3 Table — (DOCX) [file pone.0337994.s003.docx]

**Supplementary Table S3**. Frontier analysis involving SDI and periodontitis-related ASR of YLDs in 2021.

| **Location** | **SDI** | **ASR of YLDs** | **Frontier** | **Effective difference** |
| --- | --- | --- | --- | --- |
| **Nation** |  |  |  |  |
| Afghanistan | 0.34 | 53.24(21.17-112.67) | 19.60 | 33.64 |
| Albania | 0.71 | 57.31(22.61-118.78) | 11.64 | 45.67 |
| Algeria | 0.66 | 75.57(30.02-155.41) | 11.64 | 63.93 |
| American Samoa | 0.72 | 81.18(32.66-171.44) | 11.63 | 69.55 |
| Andorra | 0.87 | 64.67(25.16-137.49) | 11.63 | 53.04 |
| Angola | 0.45 | 76.58(30.73-159.14) | 11.81 | 64.76 |
| Antigua and Barbuda | 0.75 | 100.18(39.74-209.83) | 11.63 | 88.55 |
| Argentina | 0.72 | 78.87(30.95-162.88) | 11.63 | 67.25 |
| Armenia | 0.7 | 60.11(24.05-123.84) | 11.63 | 48.48 |
| Australia | 0.84 | 60.26(23.18-129.19) | 11.63 | 48.62 |
| Austria | 0.85 | 58.74(22.98-125.13) | 11.63 | 47.11 |
| Azerbaijan | 0.69 | 64.71(25.5-135.11) | 11.64 | 53.07 |
| Bahamas | 0.81 | 102.32(40.29-213.91) | 11.64 | 90.68 |
| Bahrain | 0.75 | 88.17(34.88-183.07) | 11.64 | 76.54 |
| Bangladesh | 0.49 | 124.34(48.72-252.2) | 11.63 | 112.71 |
| Barbados | 0.75 | 95.49(37.42-196.5) | 11.64 | 83.85 |
| Belarus | 0.78 | 69.39(27.7-143.1) | 11.63 | 57.75 |
| Belgium | 0.85 | 97.67(38.91-204.08) | 11.64 | 86.04 |
| Belize | 0.61 | 88.68(35.49-187.16) | 11.63 | 77.05 |
| Benin | 0.37 | 116.67(46.61-243.11) | 12.5 | 104.18 |
| Bermuda | 0.82 | 121.43(48.49-243.03) | 11.64 | 109.79 |
| Bhutan | 0.47 | 131.13(50.95-259.11) | 11.92 | 119.21 |
| Bolivia (Plurinational State of) | 0.6 | 70.83(27.43-148.07) | 11.63 | 59.2 |
| Bosnia and Herzegovina | 0.72 | 57.1(22.56-119.78) | 11.63 | 45.47 |
| Botswana | 0.64 | 28.69(10.85-63.32) | 11.64 | 17.05 |
| Brazil | 0.65 | 80.08(32.71-167.56) | 11.63 | 68.45 |
| Brunei Darussalam | 0.81 | 58.38(22.99-117.38) | 11.63 | 46.74 |
| Bulgaria | 0.77 | 47.13(18.13-99.68) | 11.63 | 35.51 |
| Burkina Faso | 0.29 | 147.69(59.41-293.79) | 25.61 | 122.07 |
| Burundi | 0.29 | 56.09(21.44-121.2) | 25.6 | 30.5 |
| Cabo Verde | 0.53 | 160.37(63.21-320.55) | 11.64 | 148.73 |
| Cambodia | 0.47 | 64.82(24.98-141.4) | 11.96 | 52.85 |
| Cameroon | 0.48 | 151.4(60.73-305.26) | 11.68 | 139.72 |
| Canada | 0.87 | 90.99(36.06-185.13) | 11.63 | 79.36 |
| Central African Republic | 0.31 | 52.36(20.35-113.3) | 21.88 | 30.48 |
| Chad | 0.24 | 112(43.97-235.56) | 41.56 | 70.44 |
| Chile | 0.77 | 92.2(36.25-191.05) | 11.63 | 80.57 |
| China | 0.72 | 70.15(27.97-144.15) | 11.63 | 58.52 |
| Colombia | 0.66 | 94.82(37.75-197.99) | 11.64 | 83.19 |
| Comoros | 0.48 | 69.88(27.29-147.1) | 11.96 | 57.92 |
| Congo | 0.58 | 74.19(29.21-154.8) | 11.64 | 62.56 |
| Cook Islands | 0.78 | 21.56(8.07-47.45) | 11.63 | 9.93 |
| Costa Rica | 0.7 | 94.63(37.44-195.44) | 11.64 | 82.99 |
| Croatia | 0.8 | 82.45(32.89-170.66) | 11.63 | 70.82 |
| Cuba | 0.67 | 90.12(36.39-187.38) | 11.63 | 78.49 |
| Cyprus | 0.84 | 57.13(21.89-120.35) | 11.64 | 45.5 |
| Czechia | 0.83 | 66.46(26.11-135.74) | 11.63 | 54.83 |
| C么te d'Ivoire | 0.43 | 120.57(47.42-246.54) | 11.94 | 108.63 |
| Democratic People's Republic of Korea | 0.57 | 42.59(16.61-90.73) | 11.63 | 30.96 |
| Democratic Republic of the Congo | 0.38 | 54.6(20.81-119.03) | 12.36 | 42.23 |
| Denmark | 0.9 | 136.17(53.79-272.21) | 11.64 | 124.53 |
| Djibouti | 0.49 | 75.78(29.25-159.02) | 11.63 | 64.15 |
| Dominica | 0.75 | 92.12(36.53-192.98) | 11.63 | 80.49 |
| Dominican Republic | 0.62 | 93.71(37.26-194.79) | 11.63 | 82.07 |
| Ecuador | 0.66 | 78.32(30.95-166.53) | 11.63 | 66.68 |
| Egypt | 0.61 | 72.98(28.62-152.51) | 11.63 | 61.36 |
| El Salvador | 0.56 | 86.17(34.13-179.33) | 11.64 | 74.53 |
| Equatorial Guinea | 0.66 | 90.43(35.68-187.85) | 11.63 | 78.8 |
| Eritrea | 0.4 | 65.22(25.91-138.45) | 11.67 | 53.54 |
| Estonia | 0.84 | 74.16(29.51-150.57) | 11.63 | 62.52 |
| Eswatini | 0.59 | 41.38(16.04-89.56) | 11.63 | 29.75 |
| Ethiopia | 0.36 | 115.52(46.7-233.38) | 19.33 | 96.19 |
| Fiji | 0.68 | 16.6(6.21-36.41) | 11.64 | 4.97 |
| Finland | 0.86 | 88.01(34.56-180.29) | 11.63 | 76.38 |
| France | 0.84 | 45.95(17.49-97.26) | 11.63 | 34.32 |
| Gabon | 0.63 | 86.35(33.98-180.64) | 11.63 | 74.72 |
| Gambia | 0.41 | 163.66(65.18-327.93) | 11.84 | 151.82 |
| Georgia | 0.73 | 62.09(24.9-127.42) | 11.63 | 50.46 |
| Germany | 0.9 | 107.16(42.5-222.34) | 11.63 | 95.53 |
| Ghana | 0.56 | 156.01(63.09-310.36) | 11.63 | 144.38 |
| Greece | 0.79 | 57.12(22.35-120.27) | 11.63 | 45.49 |
| Greenland | 0.83 | 72.77(28.85-153.52) | 11.63 | 61.15 |
| Grenada | 0.67 | 94.68(37.08-199.25) | 11.63 | 83.05 |
| Guam | 0.8 | 23.68(8.9-51.38) | 11.63 | 12.05 |
| Guatemala | 0.54 | 84.25(33.34-176.56) | 11.64 | 72.62 |
| Guinea | 0.34 | 146.79(58.97-292.45) | 19.52 | 127.27 |
| Guinea-Bissau | 0.35 | 110.99(43.79-234.16) | 19.36 | 91.63 |
| Guyana | 0.65 | 88.44(34.81-185.61) | 11.64 | 76.8 |
| Haiti | 0.45 | 70.1(27.36-146.93) | 11.86 | 58.25 |
| Honduras | 0.51 | 80.86(31.54-166.87) | 11.63 | 69.23 |
| Hungary | 0.79 | 29.9(11.28-65.87) | 11.63 | 18.28 |
| Iceland | 0.88 | 60.02(23.3-128.34) | 11.63 | 48.39 |
| India | 0.58 | 109.07(44.92-219.72) | 11.64 | 97.43 |
| Indonesia | 0.66 | 88.94(35.44-187.06) | 11.63 | 77.31 |
| Iran (Islamic Republic of) | 0.7 | 80.29(32.35-167.97) | 11.63 | 68.66 |
| Iraq | 0.66 | 75.7(29.91-157.9) | 11.63 | 64.07 |
| Ireland | 0.87 | 35.92(13.71-77.1) | 11.63 | 24.3 |
| Israel | 0.81 | 56.31(21.92-121.45) | 11.64 | 44.67 |
| Italy | 0.81 | 60.67(23.9-125.7) | 11.64 | 49.04 |
| Jamaica | 0.68 | 89.9(35.89-189.28) | 11.63 | 78.27 |
| Japan | 0.87 | 59.78(23.5-121.91) | 11.63 | 48.15 |
| Jordan | 0.73 | 72.3(28.22-149.02) | 11.63 | 60.67 |
| Kazakhstan | 0.73 | 66.26(26.43-136.49) | 11.63 | 54.63 |
| Kenya | 0.52 | 116.14(45.59-236.06) | 11.64 | 104.5 |
| Kiribati | 0.53 | 11.83(4.45-25.64) | 11.63 | 0.2 |
| Kuwait | 0.85 | 91.65(36.7-188.68) | 11.63 | 80.02 |
| Kyrgyzstan | 0.6 | 51.76(20.46-110.88) | 11.63 | 40.13 |
| Lao People's Democratic Republic | 0.49 | 37.11(14.03-79.71) | 11.64 | 25.48 |
| Latvia | 0.83 | 74.18(29.43-151.96) | 11.64 | 62.54 |
| Lebanon | 0.74 | 75.32(29.86-155.39) | 11.63 | 63.69 |
| Lesotho | 0.51 | 32.26(12.34-70.14) | 11.63 | 20.63 |
| Liberia | 0.35 | 138.37(55.69-276.77) | 19.83 | 118.54 |
| Libya | 0.73 | 71.41(28.33-147.61) | 11.64 | 59.77 |
| Lithuania | 0.86 | 75.06(29.73-153.76) | 11.63 | 63.43 |
| Luxembourg | 0.88 | 65.16(25.62-138.42) | 11.63 | 53.53 |
| Madagascar | 0.4 | 26.44(9.87-56.82) | 11.71 | 14.73 |
| Malawi | 0.38 | 60.61(23.67-131.77) | 12.33 | 48.28 |
| Malaysia | 0.74 | 50.36(19.58-107.79) | 11.63 | 38.73 |
| Maldives | 0.65 | 49.86(19.1-105.12) | 11.63 | 38.23 |
| Mali | 0.27 | 148.28(59.65-295.27) | 33.92 | 114.36 |
| Malta | 0.8 | 59.34(23.22-124.47) | 11.63 | 47.71 |
| Marshall Islands | 0.57 | 13.36(4.94-29.43) | 11.64 | 1.72 |
| Mauritania | 0.5 | 120.38(47.41-246.03) | 11.63 | 108.75 |
| Mauritius | 0.72 | 48(18.44-103.21) | 11.63 | 36.37 |
| Mexico | 0.66 | 93.9(38.21-189.96) | 11.64 | 82.26 |
| Micronesia (Federated States of) | 0.59 | 13.12(4.92-28.17) | 11.63 | 1.49 |
| Monaco | 0.91 | 78.95(31.31-163.74) | 11.64 | 67.31 |
| Mongolia | 0.62 | 59.99(23.8-125.25) | 11.64 | 48.35 |
| Montenegro | 0.8 | 60.2(23.53-123.64) | 11.65 | 48.55 |
| Morocco | 0.56 | 90.86(36.72-190.48) | 11.63 | 79.23 |
| Mozambique | 0.33 | 61.53(23.96-131.6) | 19.34 | 42.2 |
| Myanmar | 0.53 | 43.45(16.58-94.57) | 11.64 | 31.81 |
| Namibia | 0.62 | 41.62(15.93-89.53) | 11.64 | 29.98 |
| Nauru | 0.63 | 17.04(6.4-37.88) | 11.63 | 5.41 |
| Nepal | 0.43 | 81.96(31.44-172.09) | 11.67 | 70.29 |
| Netherlands | 0.89 | 57.54(22.54-121.32) | 11.64 | 45.9 |
| New Zealand | 0.85 | 85.77(34.01-180.8) | 11.63 | 74.15 |
| Nicaragua | 0.52 | 82.43(32.81-174.18) | 11.63 | 70.8 |
| Niger | 0.17 | 50.8(19.45-107.46) | 41.68 | 9.12 |
| Nigeria | 0.5 | 35.11(13.27-75.76) | 11.63 | 23.48 |
| Niue | 0.73 | 17.6(6.59-38.41) | 11.63 | 5.97 |
| North Macedonia | 0.75 | 58.15(22.87-122.13) | 11.63 | 46.52 |
| Northern Mariana Islands | 0.77 | 21.03(7.84-45.46) | 11.64 | 9.39 |
| Norway | 0.92 | 103.76(40.87-215.5) | 11.63 | 92.13 |
| Oman | 0.77 | 85.78(34.05-175.01) | 11.63 | 74.16 |
| Pakistan | 0.5 | 139.47(55.19-277.83) | 11.63 | 127.84 |
| Palau | 0.75 | 17.98(6.61-39.11) | 11.63 | 6.35 |
| Palestine | 0.63 | 64.49(25.67-133.85) | 11.63 | 52.87 |
| Panama | 0.71 | 99.57(39.25-206.31) | 11.63 | 87.94 |
| Papua New Guinea | 0.42 | 13.34(4.96-29.54) | 11.65 | 1.69 |
| Paraguay | 0.64 | 77.92(31.14-161.54) | 11.63 | 66.29 |
| Peru | 0.66 | 75.15(29.43-159.74) | 11.63 | 63.53 |
| Philippines | 0.65 | 24.88(9.74-51.05) | 11.63 | 13.25 |
| Poland | 0.81 | 77.17(30.8-159.89) | 11.63 | 65.54 |
| Portugal | 0.74 | 54.88(21.07-114.66) | 11.63 | 43.25 |
| Puerto Rico | 0.83 | 105.53(41.4-220.98) | 11.63 | 93.9 |
| Qatar | 0.85 | 99.29(38.83-204.8) | 11.63 | 87.65 |
| Republic of Korea | 0.89 | 53.48(21.12-111.01) | 11.63 | 41.85 |
| Republic of Moldova | 0.73 | 59.39(23.17-123.98) | 11.63 | 47.76 |
| Romania | 0.77 | 63.08(24.84-131.64) | 11.63 | 51.45 |
| Russian Federation | 0.81 | 70.92(28.37-146.14) | 11.64 | 59.28 |
| Rwanda | 0.44 | 67.62(26.2-144.3) | 11.88 | 55.74 |
| Saint Kitts and Nevis | 0.75 | 101.02(40.11-211.96) | 11.63 | 89.39 |
| Saint Lucia | 0.67 | 93.66(36.54-194.27) | 11.63 | 82.03 |
| Saint Vincent and the Grenadines | 0.64 | 92.37(36.75-190.86) | 11.63 | 80.74 |
| Samoa | 0.59 | 14.78(5.51-32.1) | 11.64 | 3.14 |
| San Marino | 0.89 | 61.74(24.43-131.46) | 11.63 | 50.11 |
| Sao Tome and Principe | 0.51 | 121.59(47.73-251.24) | 11.64 | 109.96 |
| Saudi Arabia | 0.82 | 55.28(21.76-116.47) | 11.64 | 43.64 |
| Senegal | 0.41 | 118.8(47.11-246.28) | 11.83 | 106.97 |
| Serbia | 0.79 | 58.81(22.83-122.56) | 11.64 | 47.18 |
| Seychelles | 0.73 | 53.44(20.49-112.43) | 11.63 | 41.81 |
| Sierra Leone | 0.36 | 167.83(64.81-339.06) | 19.37 | 148.46 |
| Singapore | 0.86 | 59.99(23.28-120.73) | 11.63 | 48.36 |
| Slovakia | 0.81 | 63.8(25.11-132.13) | 11.63 | 52.17 |
| Slovenia | 0.84 | 85.79(33.64-178.73) | 11.64 | 74.16 |
| Solomon Islands | 0.43 | 12.2(4.52-26.63) | 11.78 | 0.42 |
| Somalia | 0.08 | 41.48(15.77-91.04) | 41.48 | 0 |
| South Africa | 0.68 | 36.78(14.2-80.9) | 11.64 | 25.14 |
| South Sudan | 0.28 | 65.89(25.87-139.97) | 25.68 | 40.21 |
| Spain | 0.77 | 14.90(5.6-31.82) | 11.63 | 3.26 |
| Sri Lanka | 0.7 | 48.37(18.47-104.3) | 11.63 | 36.74 |
| Sudan | 0.54 | 57.37(22.33-124.6) | 11.64 | 45.73 |
| Suriname | 0.63 | 94.8(37.19-200.23) | 11.63 | 83.18 |
| Sweden | 0.89 | 68.92(27.44-140.28) | 11.63 | 57.3 |
| Switzerland | 0.93 | 63.55(25.17-132.67) | 11.63 | 51.92 |
| Syrian Arab Republic | 0.62 | 63.85(25.15-133.63) | 11.63 | 52.23 |
| Taiwan (Province of China) | 0.87 | 62.47(24.22-131.82) | 11.63 | 50.84 |
| Tajikistan | 0.54 | 49.75(19.63-105.63) | 11.63 | 38.12 |
| Thailand | 0.68 | 77.3(30.51-159.31) | 11.63 | 65.67 |
| Timor-Leste | 0.44 | 41.52(15.82-92.41) | 11.69 | 29.83 |
| Togo | 0.41 | 110.87(44.39-231.92) | 11.72 | 99.15 |
| Tokelau | 0.69 | 15.58(5.78-34.15) | 11.63 | 3.95 |
| Tonga | 0.63 | 14.9(5.53-32.15) | 11.64 | 3.26 |
| Trinidad and Tobago | 0.77 | 104.11(41.05-215.28) | 11.63 | 92.48 |
| Tunisia | 0.68 | 74.98(29.59-154.96) | 11.64 | 63.34 |
| Turkey | 0.71 | 85.02(33.96-177.42) | 11.63 | 73.39 |
| Turkmenistan | 0.68 | 65.82(25.78-135.37) | 11.64 | 54.18 |
| Tuvalu | 0.58 | 13.53(4.98-28.98) | 11.63 | 1.9 |
| Uganda | 0.42 | 67.47(26.35-142.94) | 11.77 | 55.7 |
| Ukraine | 0.76 | 64.48(25.89-134.66) | 11.63 | 52.85 |
| United Arab Emirates | 0.85 | 91.68(36.36-189.1) | 11.63 | 80.05 |
| United Kingdom | 0.86 | 36.04(13.79-77.91) | 11.64 | 24.4 |
| United Republic of Tanzania | 0.45 | 70.94(27.65-148.75) | 11.78 | 59.16 |
| United States of America | 0.86 | 58.5(23.31-119.15) | 11.64 | 46.87 |
| United States Virgin Islands | 0.82 | 109(42.64-229.48) | 11.63 | 97.37 |
| Uruguay | 0.72 | 107.9(42.59-223.8) | 11.63 | 96.27 |
| Uzbekistan | 0.66 | 58.2(22.73-122.27) | 11.63 | 46.58 |
| Vanuatu | 0.47 | 12.63(4.67-27.8) | 11.87 | 0.76 |
| Venezuela (Bolivarian Republic of) | 0.60 | 90.91(36.27-188.79) | 11.63 | 79.29 |
| Viet Nam | 0.63 | 45.5(17.51-96.72) | 11.63 | 33.87 |
| Yemen | 0.45 | 64.06(24.91-134.94) | 11.67 | 52.39 |
| Zambia | 0.51 | 73.91(28.29-156.28) | 11.63 | 62.28 |
| Zimbabwe | 0.47 | 30.31(11.6-66.8) | 11.87 | 18.44 |

Note: ASR: Age standardized rate, SDI: Socio-demographic index, UI: Uncertainty interval, YLDs: Years Lived with Disability.
