# Supplementary material for: Temporal trends and health inequalities in global, regional, and national years lived with disability of severe periodontitis from 1990 to 2021
Source: PLoS One. 2026 Feb 2;21(2):e0337994. doi: 10.1371/journal.pone.0337994 (PMC12863517; doi:10.1371/journal.pone.0337994)
Supplement: S1 File — (DOCX) [file pone.0337994.s004.docx]

**Authors contributions**

**Conceptualization:** Shuang Zhang, Si-Yu Liu

**Data curation:** Qiong Wang, Ya-Xi Suo, Yue-Qin Zhang

**Formal analysis:** Long Xie

**Project administration:** Chuan-Yu Hu, Long Xie

**Methodology:** Shuang Zhang, Si-Yu Liu, Long Xie

**Writing – original draft:** Shuang Zhang, Si-Yu Liu

**Writing – review & editing:** Chuan-Yu Hu, Long Xie

**Data Availability Statement**

Data are available in a public open access repository as follows: http://ghdx.healthdata.org/gbd-results-tool. All raw data have been uploaded to a designated online repository. They are available at the following link for easy access: https://doi.org/10.6084/m9.figshare.30768809

**Acknowledgments**

We thank the Institute for Health Metrics and Evaluation staff and its collaborators who prepared these publicly available data. All data used in this study are available from the corresponding author on a reasonable request.

**Competing interests**

The authors have declared that no competing interests exist.

**Financial Disclosure**

This study was funded by the National Natural Science Foundation of China (Grant No.82472966) and Natural Science Foundation of Hubei Province, China (Grant No.2023AFB703). The funders had a role in study design, data collection and analysis, decision to publish, or preparation of the manuscript.
